# *EPG5*-related Vici syndrome: a paradigm of neurodevelopmental disorders with defective autophagy

**DOI:** 10.1093/brain/awv393

**Published:** 2016-02-17

**Authors:** Susan Byrne, Lara Jansen, Jean-Marie U-King-Im, Ata Siddiqui, Hart G. W. Lidov, Istvan Bodi, Luke Smith, Rachael Mein, Thomas Cullup, Carlo Dionisi-Vici, Lihadh Al-Gazali, Mohammed Al-Owain, Zandre Bruwer, Khalid Al Thihli, Rana El-Garhy, Kevin M. Flanigan, Kandamurugu Manickam, Erik Zmuda, Wesley Banks, Ruth Gershoni-Baruch, Hanna Mandel, Efrat Dagan, Annick Raas-Rothschild, Hila Barash, Francis Filloux, Donnell Creel, Michael Harris, Ada Hamosh, Stefan Kölker, Darius Ebrahimi-Fakhari, Georg F. Hoffmann, David Manchester, Philip J. Boyer, Adnan Y. Manzur, Charles Marques Lourenco, Daniela T. Pilz, Arveen Kamath, Prab Prabhakar, Vamshi K. Rao, R. Curtis Rogers, Monique M. Ryan, Natasha J. Brown, Catriona A. McLean, Edith Said, Ulrike Schara, Anja Stein, Caroline Sewry, Laura Travan, Frits A. Wijburg, Martin Zenker, Shehla Mohammed, Manolis Fanto, Mathias Gautel, Heinz Jungbluth

**Affiliations:** 1 1 Department of Paediatric Neurology, Neuromuscular Service, Evelina’s Children Hospital, Guy’s and St. Thomas’ Hospital NHS Foundation Trust, London, UK; 2 2 Department of Basic and Clinical Neuroscience, IoPPN, King’s College London, London, UK; 3 3 Department of Neuroradiology, Evelina’s Children Hospital, Guy’s and St. Thomas’ Hospital NHS Foundation Trust, London, UK; 4 4 Department of Pathology, Boston Children’s Hospital, Boston MA 02115, USA; 5 5 Department of Clinical Neuropathology, King’s College Hospital, London, UK; 6 6 Randall Division for Cell and Molecular Biophysics, Muscle Signalling Section, King’s College, London, UK; 7 7 Viapath, Guy’s Hospital, London, UK; 8 8 Regional Molecular Genetics Laboratory, Great Ormond Street Hospital, London, UK; 9 9 Division of Metabolism, Department of Paediatric Medicine, Bambino Gesù Children’s Research Hospital, Rome; 10 10 Departments of Paediatrics, Faculty of Medicine and Health Sciences, United Arab Emirates University, Al-Ain, UAE; 11 11 College of Medicine, Alfaisal University, Riyadh, Saudi Arabia; 12 12 Department of Medical Genetics, King Faisal Specialist Hospital and Research Centre, Riyadh, Saudi Arabia; 13 13 Genetic and Developmental Medicine Clinic, Sultan Qaboos University Hospital, Muscat, Sultanate of Oman; 14 14 Ain Shams University Hospital, Egypt, Cairo; 15 15 Center for Gene Therapy, Nationwide Children’s Hospital, Columbus, Ohio, USA; 16 16 Center for Human and Molecular Genetics at The Research Institute at Nationwide Children's Hospital, Columbus, Ohio, USA; 17 17 Institute of Human Genetics, Rambam Health Care Campus and the Technion Faculty of Medicine, Haifa, Israel; 18 18 Metabolic Disease Unit, Meyer Children’s Hospital, Rambam Health Care Campus and the Technion Faculty of Medicine, Haifa, Israel; 19 19 Department of Nursing, University of Haifa, Haifa, Israel; 20 20 Institute of Rare Diseases, Institute of Genetics; Sheba Medical Centre, Tel Hashomer and the Sackler school of Medicine Tel Aviv University Ramat Aviv, Israel; 21 21 Division of Pediatric Neurology, University of Utah School of Medicine and Primary Children’s Medical Centre, Salt Lake City, Utah, USA; 22 22 University of Utah School of Medicine, Moran Eye Centre, Salt Lake City, Utah, USA; 23 23 Innovation Center for Biomedical Informatics, Georgetown University Medical Center, Washington DC, USA; 24 24 McKusick-Nathans Institute of Genetic Medicine, Johns Hopkins University, Baltimore, USA; 25 25 Division of Child Neurology and Metabolic Medicine, University Children’s Hospital, Heidelberg, Germany; 26 26 Department of Pediatrics, Section of Clinical Genetics and Metabolism, University of Colorado School of Medicine and Children's Hospital Colorado, Aurora, USA; 27 27 Department of Pathology, East Carolina University, Brody School of Medicine, Brody Medical Sciences Building, Greenville, NC 27834, USA; 28 28 Dubowitz Neuromuscular Centre, ICH, London, UK; 29 29 Medical Genetics Unit, School of Medicine of Ribeirao Preto, University of Sao Paulo; 30 30 Institute of Medical Genetics, University Hospital of Wales, Cardiff, UK; 31 31 Department of Paediatric Neurology, Great Ormond Street Children’s Hospital, London, UK; 32 32 University of Nebraska Medical Center and Childrens Hospital and Medical Center, Omaha, Nebraska, USA; 33 33 Greenwood Genetic Center, Greenville, South Carolina, USA; 34 34 Departments of Neurology, Royal Children's Hospital and Paediatrics, University of Melbourne, and Murdoch Childrens Research Institute, Melbourne Australia; 35 35 Victorian Clinical Genetics Services, Murdoch Childrens Research Institute Parkville, Australia; 36 36 Department of Paediatrics, University of Melbourne, Parkville, Australia; 37 37 Department of Clinical Genetics, Austin Health, Australia; 38 38 Department of Anatomical Pathology, Alfred Health, Australia; 39 39 Department of Anatomy and Cell Biology, University of Malta, Msida, Malta; 40 40 Section of Medical Genetics, Mater dei Hospital, Msida, Malta; 41 41 Pediatric Neurology, University Childrens Hospital, University of Duisburg-Essen University of Duisburg-Essen, Essen, Germany; 42 42 Department of Neonatology, University Childrens Hospital, University of Duisburg-Essen, Essen, Germany; 43 43 Dubowitz Neuromuscular Centre, Institute of Child Health and Great Ormond Street Hospital, 30 Guilford Street, London WC1N 1EH, UK; 44 44 Institute for Maternal and Child Health, IRCCS ‘Burlo Garofolo’, Trieste, Italy; 45 45 Department of Paediatrics, Academic Medical Centre, University of Amsterdam, Amsterdam, The Netherlands; 46 46 Institute of Human Genetics, University Hospital Magdeburg, Germany; 47 47 Department of Clinical Genetics, Guy’s Hospital, London, UK; 48 48 Department of Basic and Clinical Neuroscience, IoPPN, King’s College London, London, UK

**Keywords:** *EPG5*, ectopic P granules autophagy protein 5, Vici syndrome, neurodevelopment, neurodegeneration, callosal agenesis

## Abstract

Vici syndrome is a progressive neurodevelopmental multisystem disorder due to recessive mutations in the key autophagy gene *EPG5.* We report genetic, clinical, neuroradiological, and neuropathological features of 50 children from 30 families, as well as the neuronal phenotype of EPG5 knock-down in *Drosophila melanogaster*. We identified 39 different *EPG5* mutations, most of them truncating and predicted to result in reduced EPG5 protein. Most mutations were private, but three recurrent mutations (p.Met2242Cysfs*5, p.Arg417*, and p.Gln336Arg) indicated possible founder effects. Presentation was mainly neonatal, with marked hypotonia and feeding difficulties. In addition to the five principal features (callosal agenesis, cataracts, hypopigmentation, cardiomyopathy, and immune dysfunction), we identified three equally consistent features (profound developmental delay, progressive microcephaly, and failure to thrive). The manifestation of all eight of these features has a specificity of 97%, and a sensitivity of 89% for the presence of an *EPG5* mutation and will allow informed decisions about genetic testing. Clinical progression was relentless and many children died in infancy. Survival analysis demonstrated a median survival time of 24 months (95% confidence interval 0–49 months), with only a 10th of patients surviving to 5 years of age. Survival outcomes were significantly better in patients with compound heterozygous mutations (*P* = 0.046), as well as in patients with the recurrent p.Gln336Arg mutation. Acquired microcephaly and regression of skills in long-term survivors suggests a neurodegenerative component superimposed on the principal neurodevelopmental defect. Two-thirds of patients had a severe seizure disorder, placing *EPG5* within the rapidly expanding group of genes associated with early-onset epileptic encephalopathies. Consistent neuroradiological features comprised structural abnormalities, in particular callosal agenesis and pontine hypoplasia, delayed myelination and, less frequently, thalamic signal intensity changes evolving over time. Typical muscle biopsy features included fibre size variability, central/internal nuclei, abnormal glycogen storage, presence of autophagic vacuoles and secondary mitochondrial abnormalities. Nerve biopsy performed in one case revealed subtotal absence of myelinated axons. Post-mortem examinations in three patients confirmed neurodevelopmental and neurodegenerative features and multisystem involvement. Finally, downregulation of *epg5* (CG14299) in *Drosophila* resulted in autophagic abnormalities and progressive neurodegeneration. We conclude that *EPG5*-related Vici syndrome defines a novel group of neurodevelopmental disorders that should be considered in patients with suggestive features in whom mitochondrial, glycogen, or lysosomal storage disorders have been excluded. Neurological progression over time indicates an intriguing link between neurodevelopment and neurodegeneration, also supported by neurodegenerative features in *epg5*-deficient *Drosophila*, and recent implication of other autophagy regulators in late-onset neurodegenerative disease.

## Abstract (precis)

Vici syndrome is a progressive neurodevelopmental multisystem disorder caused by mutations in the autophagy gene *EPG5*. Byrne *et al.* characterise the phenotype of 50 affected children, revealing callosal agenesis, cataracts, hypopigmentation, cardiomyopathy, immune dysfunction, developmental delay and microcephaly. Downregulation of *epg5* in Drosophila results in autophagic abnormalities and progressive neurodegeneration.

## Graphical abstract

**Figure:**
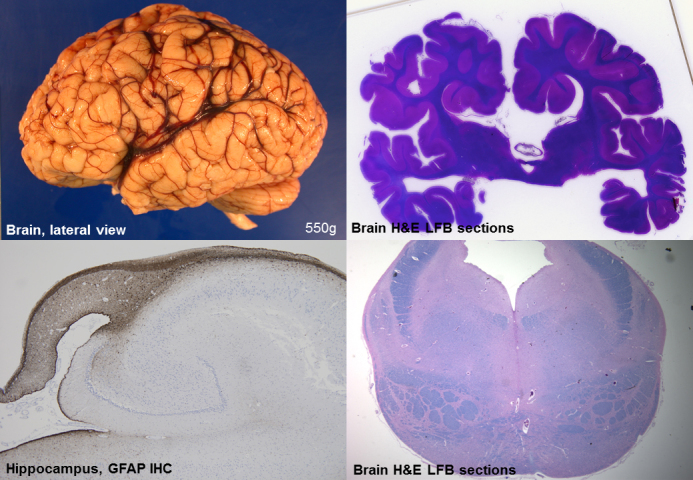
Vici syndrome is a progressive neurodevelopmental multisystem disorder caused by mutations in the autophagy gene *EPG5*. Byrne *et al.* characterise the phenotype of 50 affected children, revealing callosal agenesis, cataracts, hypopigmentation, cardiomyopathy, immune dysfunction, developmental delay and microcephaly. Downregulation of *epg5* in Drosophila results in autophagic abnormalities and progressive neurodegeneration.

## Introduction

Vici syndrome (OMIM 242840) is a severe, early-onset neurodevelopmental disorder characterized by the key features of callosal agenesis, cataracts, cardiomyopathy, generalized hypopigmentation, and combined immunodeficiency. Since its recognition as a distinct entity (Vici *et al.*, 1988), additional case reports have suggested an extended phenotype including sensorineural hearing loss, a skeletal myopathy, and other, more variable multi-systemic features (del Campo *et al.*, 1999; Chiyonobu *et al.*, 2002; Miyata *et al.*, 2007, 2014; Al-Owain *et al.*, 2010; Rogers *et al.*, 2011; Finocchi *et al.*, 2012; Ozkale *et al.*, 2012; Said *et al.*, 2012; Cullup *et al.*, 2013; Ehmke *et al.*, 2014; Filloux *et al.*, 2014).

In 2013, our team linked Vici syndrome to recessive mutations in *EPG5* on chromosome 18q (Cullup *et al.*, 2013), encoding ectopic P-granules autophagy protein 5 (EPG5) with a key role in autophagy in multicellular organisms (Tian *et al.*, 2010). Autophagy is a highly conserved lysosomal degradative pathway, with important roles in cellular homeostasis including infection defence, quality control of proteins and organelles, and metabolic adaptation (for review see Mizushima, 2007; Levine and Kroemer, 2008; Klionsky *et al.*, 2012). The autophagy pathway involves several tightly regulated steps, evolving from the initial formation of isolation membranes (or phagophores) to autophagosomes, whose fusion with lysosomes results in the final structures of degradation, autolysosomes. The EPG5 protein has been implicated in the penultimate autophagy stages in *Caenorhabditis elegans* (Tian *et al.*, 2010) and, more recently, in human cells, suggesting that EPG5 deficiency results in failure of autophagosome-lysosome fusion (Cullup *et al.*, 2013) and, ultimately, impaired cargo delivery to the lysosome.

Implication of the autophagy pathway in early-onset neurodevelopmental disorders such as Vici syndrome, Niemann-Pick type C and Lafora disease (Ebrahimi-Fakhari *et al.*, 2014), and adult-onset neurodegenerative disorders such as dementia, amyotrophic lateral sclerosis, and Parkinson’s disease (Nixon, 2013) suggests an intriguing link between these groups of conditions and emphasizes the crucial importance of normally functioning autophagy for both neuronal formation and maintenance. A particular link between EPG5 deficiency, neurodevelopment, and neurodegeneration is also suggested by the observation of defective autophagy and neurodegenerative features resembling human ALS in the *Epg5* knockout mouse (Zhao *et al.*, 2013*a*, *b*).

The relative frequency of specific clinical features, natural history, overall prognosis, and genotype–phenotype correlations have not yet been reported for *EPG5*-related Vici syndrome.

Here we report genetic, clinical, neuroradiological, and neuropathological features of 50 patients from 30 unrelated families with *EPG5*-related Vici syndrome (including 33 previously unreported cases). To further investigate the functional effects of EPG5 deficiency on autophagy and neuronal function, we studied the effects of conditional EPG5 knockdown in neurons of the fruit fly, *Drosophila melanogaster*, a widely used model for the study of neurodegenerative disease (Marsh and Thompson, 2006).

## Materials and methods

### Patients

Patients were identified over a 3-year period through the diagnostic service for *EPG5*-related Vici syndrome and related disorders at Guy’s Hospital, Guy’s and St Thomas’ NHS Foundation Trust, UK, and other participating centres offering diagnostic *EPG5* testing. Inclusion criteria for diagnostic *EPG5* testing at the Guy’s and St. Thomas’ diagnostic laboratory were patients with ‘Vici syndrome’ (defined by the presence of at least four out of the five key features, i.e. callosal agenesis, cataracts, cardiomyopathy, hypopigmentation and combined immunodeficiency) and patients with ‘Vici-like syndromes’ (defined by the presence of three or fewer of the five key features) as documented on our referral form (Supplementary Data). Additional inclusion criteria for this study were identification of at least one pathogenic mutation in the *EPG5* gene (confirmed Vici syndrome), or presence of the clinical phenotype and genetic confirmation of an *EPG5* mutation in a relative with similar features (probable Vici syndrome). The only exclusion criterion for the present *EPG5* genotype–phenotype study was the failure to identify at least one pathogenic *EPG5* mutation in patients or an affected relative.

The study was approved and performed under the ethical guidelines issued by the participating institutions for clinical studies. Parents/guardians provided written informed consent for genetic analysis and consented to use of the clinical data in anonymized forms, and to the publication of recognizable clinical photographs where applicable. Research ethics committee approval was obtained for transfer and biobank storage of specimen (blood, fibroblasts, muscle, nerve) for research purposes (REC Reference 06/Q0406/33).

Data were extracted from the questionnaire by two researchers (S.B., H.J.). Data analysis was carried out using SPSS 22. Parametric tests were performed where the data were normally distributed, and non-parametric tests were used if the data were not normally distributed. Survival analysis was carried out using univariable and multivariable survival analysis. Censor date was set as the last date that information was available on the clinical status of the patient (i.e. alive or deceased). Statistical significance was set at *P* < 0.05.

### Genetic testing

Genomic DNA was extracted from peripheral blood leucocytes according to standard procedures. Diagnostic screening for *EPG5* mutations at Guy’s Hospital, Guy’s and St Thomas’ NHS Foundation Trust, UK, was performed by bidirectional Sanger sequencing as previously described (Cullup *et al.*, 2013). Assessment of pathogenicity of novel *EPG5* variants was carried out using bioinformatics software Alamut v.2.0.

### Clinical information

This was a retrospective, cross-sectional study of all cases with a diagnosis of confirmed or probable *EPG5*-related Vici syndrome referred to our diagnostic service and other participating centres over a 3-year period. Clinical data are reported for the 38 genetically confirmed cases.

Data were obtained from case notes held at the respective referral centres and previous publications (where applicable), and collected based on the items included in the referral form for diagnostic *EPG5* sequencing in our laboratory (Supplementary Data).

### Neuroradiological features

Brain imaging was performed in 46 patients, and 18 MRI brain scans were available for review. All available MRI brain scans were reviewed and analysed by two experienced paediatric neuroradiologists (J.M.U.K., A.S.) in consensus.

### Neuropathological features

Muscle biopsies were performed in 17 children, and one patient each had a nerve (Patient 18.1), liver (Patient 16.1), bone marrow (Patient 16.1) and skin (Patient 25.1) biopsy for ultrastructural examination. Four patients underwent a post-mortem examination, three of them previously reported (Vici *et al.*, 1988; Rogers *et al.*, 2011; Miyata *et al.*, 2014). Pathological samples obtained during life and post-mortem specimens were reviewed by experienced pathologists (I.B., C.A.S., P.B., C.L.).

### Generation and characterization of a conditional *epg5 Drosophila* knock-down

*Drosophila* strains used included: *w1118*, *CG-Gal4*, *GMR-Gal4*, *Ubi-Gal80^ts^*, *UAS-Epg5^IR^*(TRiP GL00468), *UAS-GFP::Atg8a*, *UAS-mRFP*. Semi-thin sections and analysis of adult retina neurons were performed as previously reported (Nisoli *et al.*, 2010). All crosses and experiments were performed at 25°C. Third instar larvae were starved 6 h in PBS + 20% sucrose to induce autophagy. Fat bodies were dissected, mounted in Vectashield (Vectorlabs) and readily imaged on a confocal microscope (Zeiss) (Joffre *et al.*, 2015).

## Results

### General findings

This series includes 50 patients from 30 families with *EPG5*-related Vici syndrome; 33 patients from 17 families are reported here for the first time. Thirty-eight had genetically confirmed Vici syndrome and in 12 additional patients, the diagnosis of Vici syndrome was assumed based on suggestive features and confirmation of the genetic diagnosis in an affected relative. Males (*n* = 24) and females (*n* = 26) were almost equally represented.

### Genetic results

**Figure 1:**
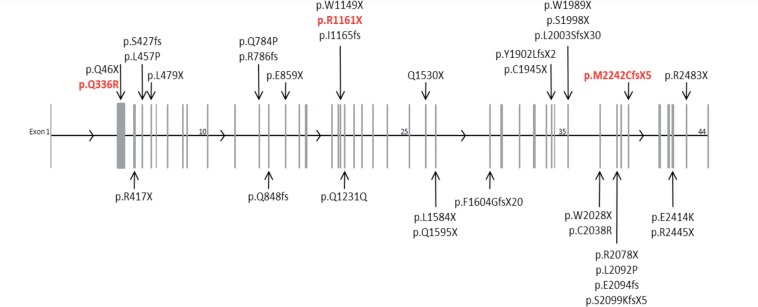
**Distribution of disease-causing mutations in *EPG5.*** The *EPG5* gene is represented, with the 44 exons depicted as grey vertical bars (exons 1, 10, 25, 35 and 44 are numbered for orientation). The exon position of Vici-causing mutations are indicated by the arrows, with the mutation details listed at the arrow tail. Mutations found in two or more unrelated patients due to possible founder effects are coloured red. EPG5 mutations/variants described according to HGVS guidelines and transcript number NM_020964.2.

We identified a total of 39 different *EPG5* mutations in 38 patients from 30 families of varying ethnicity (Table 1 and Fig. 1).

**Table 1:** *EPG5* mutations in patients with Vici syndrome

Family	Origin	Ethnicity	Consanguinity	Mutation 1			Mutation 2		
				Nucleotide	Amino acid	Exon	Nucleotide	Amino acid	Exon
1	Italy	Caucasian	No	c.4588C>T	p.Gln1530*	26	c.5704dupT	p.Tyr1902Leufs*2	33
2	Italy	Caucasian	No	c.2413-2A>G			c.6724delA	p.Met2242Cysfs*5	39
3	UK	British-Asian	No	c.1253-1G>T			c.5110-1G>C		
4	Germany	Turkish	Yes	c.4952+1G>A	p.Phe1604Glyfs*20	28	c.4952+1G>A	p.Phe1604Glyfs*20	28
5	Netherlands	Turkish	Yes	c.3481C>T	p.Arg1161*	19	c.3481C>T	p.Arg1161*	19
6	USA	Caucasian	No	c.5835T>A	p.Cys1945*	33	c.1370T>C	p.Leu457Pro	4
							c.2351A>C	p.Gln784Pro	12
7	USA	Caucasian	Yes	c.1007A>G	p.Gln336Arg	2	c.1007A>G	p.Gln336Arg	2
8	USA	Caucasian	No	c.2575G>T	p.Glu859*	14	c.6232C>T	p.Arg2078*	37
9	Saudi Arabia	Arabic	Yes	c.4751T>A	p.Leu1584*	27	c.4751T>A	p.Leu1584*	27
10	Japan	Japanese	No	c.2719-1G>A			c.6295dupA	p.Ser2099Lysfs*5	37
11	Malta	Caucasian	No	c.6724delA	p.Met2242Cysfs*5	39	c.6724delA	p.Met2242Cysfs*5	39
12	USA	Caucasian	No	c.6005_6006dupAG	p.Leu2003Serfs*30	35	c.6112T>C	p.Cys2038Arg	36
13	UAE	Arabic	Yes	c.4783C>T	p.Gln1595*	27	c.4783C>T	p.Gln1595*	27
14	Egypt	Arabic	Yes	c.2355delC	p.Arg786Glufs*10	12	c.2355delC	p.Arg786Glufs*10	12
15	Israel	Israeli-Arabic	Yes	c.5993C>G	p.Ser1998*	35	c. 5993C>G	p.Ser1998*	35
16	UK	Caucasian	No	c.1007A>G	p.Gln336Arg	2	?	?	
17	Germany	Turkish	Yes	c.1278delC	p.Ser427Leufs*6	4	c.1278delC	p.Ser427Leufs*6	4
18	Australia/Greece	Caucasian	Yes	c.7333C>T	p.Arg2445*	42	c.7333C>T	p.Arg2445*	42
19	UAE	Arabic	Yes	c.1249C>T	p.Arg417*	3	c.1249C>T	p.Arg417*	3
20	Italy	Caucasian	No	c.7447C>T	p.Arg2483*	43	c.1435_1438delCTTC	p.Leu479*	5
21	Oman	Arabic	Yes	c.6084G>A	p.Trp2028*	36	c.6084G>A	p.Trp2028*	36
22	Saudi Arabia	Arabic	Yes	c.3693G>A	p.Gln1231Gln	20	c.3693G>A	p.Gln1231Gln	20
23	Germany	Caucasian	No	c.5869+1G>A			c.5966G>A	p.Trp1989*	35
24	USA	Caucasian	No	c.136C>T	p.Gln46*	2	c.6275T>C	p.Leu2092Pro	37
25	Brazil	Caucasian	No	c.3481C>T	p.Arg1161*	19	c.6280delG	p.Glu2094Lysfs*23	37
26	Israel	Ashkenazi	Yes	c.1007A>G	p.Gln336Arg	2	c.1007A>G	p.Gln336Arg	2
27	USA	Caucasian	No	c.2542delC	p.Gln848Argfs*25	13	c.3493_3497delATCCT	p.Ile1165Leufs*8	19
28	Israel	Israeli-Arabic	Yes	c.3447C>T	p.Trp1149*	19	c.3447C>T	p.Trp1149*	19
29	USA	Ashkenazi	No	c.1007A>G	p.Gln336Arg	2	c.1007A>G	p.Gln336Arg	2
30	USA	Caucasian	No	c.1249C>T	pArg417*	3	c.7240G>A	p.Glu2414Lys	42

*EPG5* mutations/variants described according to HGVS guidelines and transcript reference NM_020964.2. Stop mutations are indicated by an asterisk. *EPG5* mutations in Families 1–13 have been previously reported in Cullup *et al.* (2013), *EPG5* mutations in Families 14–30 are reported here for the first time. In Family 16, only one heterozygous pathogenic *EPG5* variant was identified in the proband. The splicing change indicated in Family 4 was derived from cDNA sequencing. The c.3693G>A (p.Gln1231Gln) sequence variant identified in Family 22 occurs at the last base of the exon and is expected to cause aberrant splicing of the *EPG5* transcript.

Nineteen of these mutations have not been previously reported. In 12 additional affected cases from the same families, the *EPG5* genotype was assumed, based on the criteria outlined above. None of the novel *EPG5* mutations identified were found on publically available databases of human genetic variation (dbSNP version 135, www.1000genomes.org/; 1000 Genomes database, www.ncbi.nlm.nih.gov/projects/SNP/; Exome Variant Server, evs.gs.washington.edu/EVS/), except for *EPG5* p.Gln336Arg documented in one heterozygous individual on the NCBI SNP database. Where performed, co-segregation studies were compatible with recessive inheritance. Twenty-one of 38 (55%) patients were homozygous and 17/38 (45%) of patients were compound heterozygous for *EPG5* mutations. While biallelic inheritance was thus confirmed in 98% of patients with *EPG5*-related Vici syndrome, in one case, included because of diagnostic clinical features (Patient 16.1), only one heterozygous but clearly pathogenic *EPG5* mutation was identified. Copy number testing was not performed in this patient. The vast majority of *EPG5* mutations were predicted to result in a truncated EPG5 protein, with only a small number of missense mutations identified. Most mutations were private, with the exception of three recurrent mutations, p.Met2242CysfsX5 identified in the heterozygous state in an Italian and in the homozygous state in an unrelated Maltese patient without known parental consanguinity, p.Arg417X identified in the homozygous state in a patient from the Middle East and in the heterozygous state in a Caucasian child from the USA, and p.Gln336Arg identified in the homozygous (*n* = 3) and in the heterozygous (*n* = 1) state in four unrelated patients with definite or possible Ashkenazi ancestry.

### Family history

Parental consanguinity was reported in 43% (13/30) of families, corresponding to the large number of homozygous *EPG5* mutations identified in our cohort. A family history of suspected Vici syndrome (or an undiagnosed multisystem disorder with suggestive features) in an already deceased relative was reported in nine families before the genetic diagnosis in the index case had been established. We also noted a high frequency of twin pregnancies, with three probands having a family history of multiple births, and two sets of affected children being twins (one set identical, one set fraternal).

In families currently still under active follow-up (*n* = 22 families) we obtained more detailed histories (Supplementary Data), which suggested a relatively high incidence of cancer. A first-degree relative of one of the *EPG5*-mutated patients developed early-onset cataracts, and vitiligo was occasionally reported. There was also a history of neurodegenerative disorders, in particular Parkinson’s disease, in 2 of 22 families under ongoing follow-up.

### Clinical features

**Figure 2:**
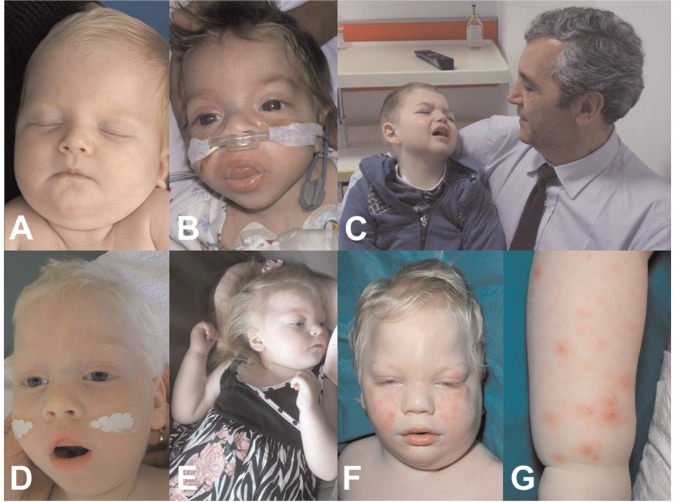
**Clinical features of *EPG5*-related Vici syndrome.** Clinical photographs from Patient 5.2 (**A**), Patient 3.1 (**B**), Patient 2.1 (**C**), shown with Professor Carlo Dionisi Vici, the original describer of Vici syndrome, Patient 23.1 (**D**), Patient 24.2 (**E**) and Patient 16.1 (**F** and **G**) at different ages. There is marked hypopigmentation, never absolute but always relative to the familial and ethnic background [Patient 5.2 (**A**) and Patient 3.1 (**B**) were of Turkish and British-Indian parentage, respectively]. Cataracts may be either present from birth (**B**, note reduced red reflex in the right eye) or develop over the first year of life. Some patients may have myopathic facial features (**D**, note tent-shaped mouth) and/or other clinical signs of a skeletal muscle myopathy. Few patients may have coarse facial features reminiscent of a lysosomal storage disorder, either present from birth (**B**) or developing with increasing age. Although head circumference is consistently normal at birth, all patients with *EPG5*-related Vici syndrome ultimately manifest microcephaly over time (**C** and **E**). In some patients, a recurrent, often confluent maculo-papular rash (**F** and **G**) was noted.

The main clinical findings and their relative frequencies in genetically confirmed cases with *EPG5*-related Vici syndromes are summarized in Table 2 and outlined in more detail below, and in Supplementary Data. Typical clinical features are illustrated in Fig. 2.

**Table 2:** Clinical features of *EPG5*-related Vici syndrome

Feature	%
**Principal diagnostic features**	
Absent corpus callosum^a^	100
Gross developmental delay^b^	100
Immune problems^a^	100
Failure to thrive^b^	97
Pale skin/light hair^a^	95
Microcephaly^b^	90
Cardiomyopathy^a^	82
Cataracts^a^	76
**Other prominent features**	
Neonatal presentation	87
Seizures	59

Clinical features and their relative frequencies compiled from genetically confirmed cases of Vici syndrome in this series (*n* = 38).

^a^Original diagnostic feature.

^b^New diagnostic feature identified in this series.

#### Perinatal history

Presentation was mainly neonatal with profound hypotonia and associated respiratory and bulbar involvement. Some mothers had reported reduced foetal movements. Few affected neonates had arthrogryposis (*n* = 6), and/or cleft palate (*n* = 3), and/or minor dysmorphic features such as second and third toe syndactyly (*n* = 2). Head circumference and weight were within the expected centiles for gestational age at birth but both progressively declined over the course of the first year of life (Supplementary Data).

#### Developmental history

All children were profoundly delayed. Most children did not achieve head control. Only four children learned to roll over; all four subsequently lost this skill. None achieved independent sitting. Development of social and communication skills was also profoundly delayed, with some affected infants acquiring social smile; those who did subsequently lost this skill. Speech was universally absent, although a number of children could make sounds. Two of the older children were reported to be able to answer simple yes/no questions with gestural responses.

#### General features

Failure to thrive was virtually universal, with weight within the normal range at birth (2nd to 75th centile), but subsequently dropping below the third centile despite adequate caloric intake. Most children required gastrostomy tube feeding. Fewer than 10% of children gained a normal body weight despite adequate feeding with parenteral nutrition.

#### Neurological and neuromuscular features

An associated seizure disorder was present in 59% of cases where information was available (20/34). Seizures comprised early-onset epileptic encephalopathies with burst-suppression on EEG and infantile spasms with EEG features compatible with hypsarrhythmia and, with few exceptions, were difficult to control despite exhaustive treatment trials with various anticonvulsants. Seizure types varied from apnoeic seizures, to myoclonic jerks, to tonic-clonic seizures. The epilepsy syndrome was described as ‘Lennox-Gastaut-like’ in two cases. EEG discharges were often multifocal. Where epilepsy was a feature, onset was within the first year of life.

Marked hypotonia with associated generalized weakness, paucity of movement, gross motor developmental delay, and mildly but consistently elevated creatine kinase (CK) levels (median 450 IU/l; IQR 420–930 IU/l; range 314–1400 IU/l) suggested a concomitant skeletal myopathy, confirmed in 17 patients on pre-mortem muscle biopsy (see below). Deep tendon reflexes were reduced or absent in most cases. Seven patients had EMG/nerve conduction studies, six of which demonstrated evidence of a myopathy.

Despite a normal head circumference at birth, microcephaly was subsequently reported in 90% of children where measurements were available (28/31) (Supplementary Data). One older patient showed features of an evolving dystonic-choreoathetoid movement disorder and in another a spastic quadriparesis was diagnosed, although the latter may have been related to a secondary hypoxic insult.

#### Ocular and auditory features

Seventy-six per cent (29/38 genetically confirmed cases) of patients had cataracts, either at birth or evolving over the first months of life. Optic atrophy and retinal changes were present in 11 and 14 patients, respectively. Five children had ocular albinism. Ten patients had formal neurophysiological visual assessment by visual evoked potential/electroretinogram, of which almost all (8/10) were abnormal. Neuro-ophthalmological features from one patient have been previously reported in detail (Filloux *et al.*, 2014), and included retinal hypopigmentation, poor foveal development, b-wave amplitudes in the lower normal range on ERG, and probable misrouting at the optic chiasm similar to what has been described in typical albinism. Brain auditory evoked responses provided evidence for sensorineural hearing impairment in six of nine patients where performed.

### Additional multisystem involvement

#### Heart

Most children had a hypertrophic and/or dilated cardiomyopathy (82%, 28/34); this was not always present in the first months of life, however, the cardiomyopathy appeared to be progressive where consecutive cardiac ultrasounds were performed (Supplementary Data). Intermittent deterioration of cardiac function associated with intercurrent illness was also observed. Structural congenital heart abnormalities comprising patent foramen ovale, ventricular or atrial septal defects, hypoplastic aortic arch or mitral valve insufficiency were found in 10 children.

#### Skin

Generalized skin and hair hypopigmentation was present in most patients, always relative to familial and ethnic background. In a number of children (*n* = 6), unexplained intermittent maculo-papular rashes were also noted.

#### Immune system

All patients (38/38) had recurrent and unusual infections suggestive of an underlying immunodeficiency, most frequently recurrent pneumonia. Infective agents cultured included *Streptococcus viridans*, *Staphylococcus hominis*, *Klebsiella*, *Pseudomonas*, *and Candida albicans*. In addition, recurrent unexplained pyrexias without identification of a pathogenic organism were also a feature. A large abscess developed after varicella vaccination in one child, requiring surgery. Where formal assessment of immune function was performed (*n* = 11), this suggested a combined immunological defect with both T and B cells being affected. One child had normal immunological studies, another (Patient 25.1) was diagnosed with severe combined immunodeficiency. In four cases intravenous immunoglobulin was reported to be of benefit (Finocchi *et al.*, 2012). Haematological abnormalities were present in 24 patients, with anaemia, and leucopaenia commonly reported.

#### Other organ systems

Variable additional organ involvement concerned thyroid, thymus, lungs, liver, and kidneys and comprised both defects of organ formation (e.g. thyroid agenesis, thymus aplasia, pulmonary hypoplasia) and function (e.g. hypothyroidism, electrolyte disturbance). A peculiar feature in six patients was intermittent episodes of profound electrolyte disturbance (mainly hypokalaemia) suggestive of renal tubular dysfunction, as reported previously in one case (Miyata *et al.*, 2007).

#### Progression and prognosis

Vici syndrome is a severe, life-limiting condition. Survival analysis demonstrated that the median survival time was 24 months [95% confidence interval (CI) 0–39 months], with only a tenth of patients surviving to 5 years of age. Twelve patients were still alive at the censor date, and the oldest living patient is 10 years old. Cardiorespiratory failure in the context of a respiratory tract infection and/or immunodeficiency was indicated as the most common cause of death.

### Genotype–phenotype correlations

As most of the *EPG5* mutations identified were distributed throughout the entire *EPG5* coding sequence and private to individual families, precise genotype–phenotype correlations are difficult to establish. However, analysing survival time until death or censor date, we could establish that patients with homozygous mutations died at a median age of 9 months compared to 48 months in patients with heterozygous *EPG5* mutations (*P* = 0.046) (Supplementary Data). Compared to patients with truncating *EPG5* mutations, patients homozygous (*n* = 3) or compound heterozygous (*n* = 1) for the recurrent p.Gln336Arg mutation, one of the few missense mutations identified in our cohort, had a longer life expectancy with a median survival of 78 months. Whether this observation is due to the expression of a missense mutant protein conferring residual function, or partial impairment of *EPG5* splicing (p.Gln336 is the last amino acid of its exon and the mutation c.1007A>G is predicted to abolish the donor splice site) (Cullup *et al.*, 2013) is currently uncertain. Interestingly, two of the patients with the p.Gln336Arg mutation had not developed cataracts, and three did not have cardiomyopathy at the censor date.

Binary logistic regression was used to compare the number of eight key features identified (absent corpus callosum, cataracts, hypopigmentation, cardiomyopathy, immune dysfunction, profound developmental delay, progressive microcephaly, failure to thrive), with the likelihood of a positive *EPG5* genetic test in all 66 patients tested (36 *EPG5*-positive, 30 *EPG5*-negative), indicating that the presence of all eight features had a specificity of 97%, and a sensitivity of 89% for a positive *EPG5* genetic test.

### Neuroradiological features

**Figure 3:**
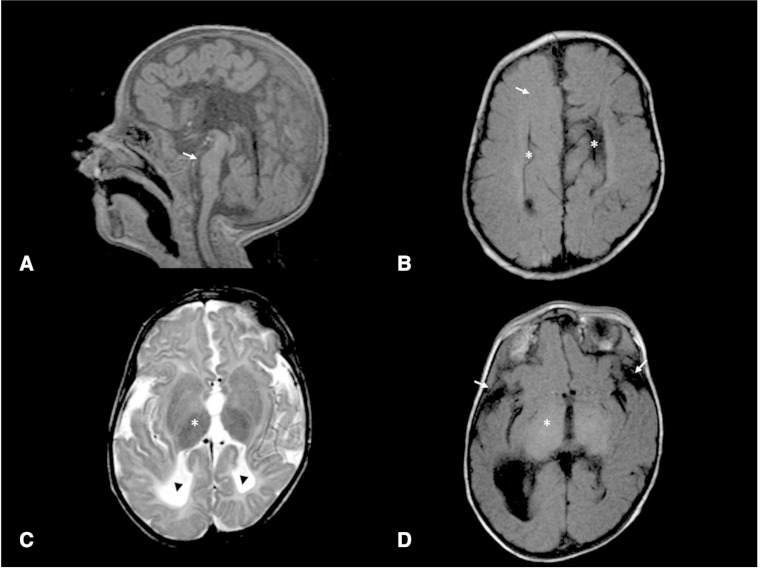
**Radiological features of *EPG5*-related Vici syndrome.** (**A**) Midline sagittal T_1_ sequence showing complete agenesis of the corpus callosum. There is moderate-to-severe hypoplasia of the pons (arrow). In contrast, the cerebellar vermis appears well formed. (**B**) Axial T_1_ image at level of the body of the lateral ventricles show parallel configuration of the lateral ventricles (asterisk), typical for callosal agenesis. Moreover, the expected high T_1_ signal of myelin within the white matter is generally reduced (arrow), in keeping with delay in myelination. (**C** and **D**) Axial T_1_ and T_2_ at the level of the thalami show diffuse abnormal low T_2_ and high T_1_ signal within the thalamus (asterisk), a feature which has been reported in lysosomal storage disorders, but was only seen in a small proportion of our cases. There is also reduction in white matter bulk and reduced opercularization of Sylvian fissures (arrows). Note is made of colpocephaly with prominence of posterior horns of lateral ventricles (arrowheads), a feature seen with callosal agenesis.

Brain MRI scans were available for review from 18 children in our series with *EPG*-related Vici syndrome. Key neuroradiological features are illustrated in Fig. 3 and summarized in Supplementary Data.

Complete agenesis of the corpus callosum was present on all MRI scans reviewed and was also reported on brain MRI, CT, and/or cranial ultrasound in the remaining patients for whom scans were unavailable for review. Colpocephaly was consistently associated with callosal agenesis, often with asymmetric dilation of the posterior aspect of the lateral ventricles. The pons invariably appeared hypoplastic relative to the cerebellum and remainder of the brainstem. The cerebellar vermis was generally well formed, with only two patients showing minor deficiency of the inferior vermis. Mild prominence of the cisterna magna was seen in few cases but other recognized cystic malformations of the posterior fossa were not observed. Five patients showed diffusely abnormal low T_2_ signal within the thalami, with additional diffuse T_1_ hyperintensity in one of those cases. Thalamic changes were associated with older age at MRI (median age of 27 months in those with, compared to 8.5 months in those without thalamic changes, *P* = 0.04). The basal ganglia were otherwise normally formed in all patients. Neuromigrational abnormalities were not a significant feature, although two patients showed mild simplification of sulcation pattern for age. Myelination was delayed for age in all patients. White matter bulk was reduced and the Sylvian fissures showed reduced opercularization to varying degrees in all patients.

### Neuropathological features

Ante-mortem muscle biopsies performed in 17 patients revealed a consistently associated myopathy (Supplementary Data), on light microscopy characterized by (in order of decreasing frequency): increased variability in fibre size, increased internalized and/or centralized nuclei, type 1 atrophy (consistent in some with designation as fibre type disproportion), increased glycogen storage, type one predominance, and cores. There was variably increased acid phosphatase staining localizing to vacuoles, and reddish staining of some atrophic fibres on the Gomori trichrome stain, suggesting localized basophilia or mitochondrial abnormalities. Occasionally, mild dystrophic features with increase in connective tissue and few regenerating and angular fibres were noted. Where specific immunohistochemical studies were performed, increase in autophagy marker proteins (p62, LAMP2) in the region of the vacuoles was observed. Respiratory chain enzyme studies performed in seven patients were normal in four but showed variable reduction of respiratory chain complexes (except complex II) in the other three.

**Figure 4:**
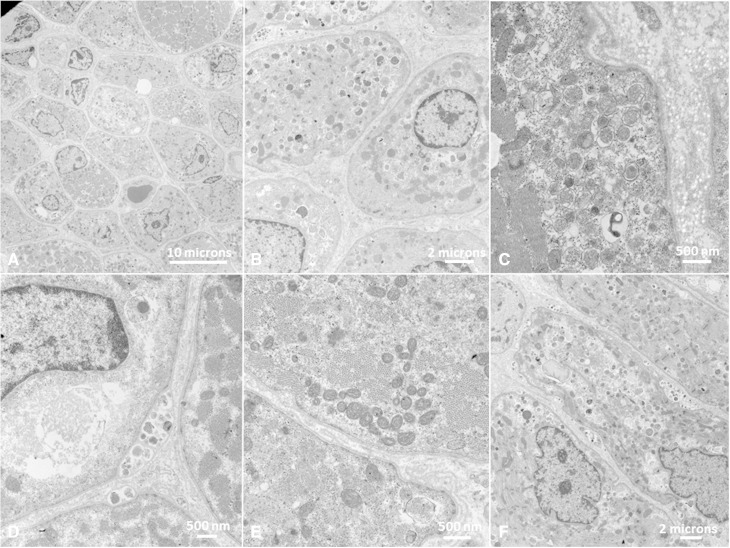
**Ultrastructural abnormalities of muscle in *EPG5*-related Vici syndrome.** Muscle biopsies from Patients 4.1 (**A–B** and **D–F**) and 27.1 (**C**). On electron microscopy, variability in fibre diameter (**A**) and central nuclei (**A** and **B**) often surrounded by a ‘ring-shaped’ mitochondrial arrangement (**B**) were noted. There was abundant intracellular debris (**C**), most commonly membrane-bound, often deposited within basal lamina layers (**D**) and with evidence of ongoing exocytosis. There was an increase in both free and membrane-bound glycogen in lysosomes (**C** and **E**). There was generalized reduction of often poorly formed myofilaments, admixed with deposited abnormal material (**F**).

Ultrastructural examination (Fig. 4) performed in 12 patients confirmed central localization of nuclei in many fibres, corresponding to findings on light microscopy. There were variable degrees of subsarcolemmal accumulation of vacuoles, glycogen and mitochondria. Deposition of debris within basal lamina layers resembling ultrastructural findings in X-linked myopathy with excessive autophagy (MEAX) (Ramachandran *et al.*, 2013) was commonly observed, often with evidence of ongoing exocytosis. Nuclei were often central and surrounded by a ‘ring-shaped’ mitochondrial arrangement, similar to what has been described in *DNM2*-related centronuclear myopathy (CNM) (Susman *et al.*, 2010). Mitochondria often showed abnormal size, shape, and internal structure. There was generalized reduction of often poorly formed myofilaments. Sarcomeric disarrangements resembling minicores were seen in one patient.

Sural nerve biopsy performed in one patient showed almost complete absence of myelinated axons (Supplementary Data).

Skin biopsy performed for ultrastructural examination in one patient showed a reduced number of melanosomes with a low degree of melanization but no other abnormalities. Other findings were considered suggestive of neuraxonal dystrophy as previously reported (Ozmen *et al.*, 1991). Ante-mortem bone marrow performed in one patient was normal.

**Figure 5:**
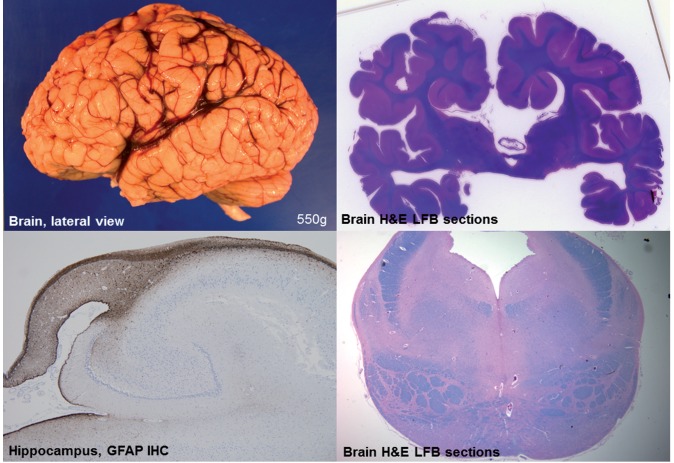
**Neuropathological features of *EPG5*-related Vici syndrome.** (**A**) Gross lateral view of left hemisphere. Note somewhat indistinct gyral pattern, and relatively prominent sulci for age. The insula is visible, consistent with an opercularization defect, and the Sylvian fissure extends more posteriorly than normal. (**B**) Whole-brain section stained with Luxol Fast blue/haematoxylin and eosin (LFB/H&E) at the level of the thalamus. Note the callosal agenesis, prominent temporal ventricles, and malrotated hippocampi. (**C**) Hippocampus immunostained for glial fibrillary acidic protein (GFAP). Most notable is the diminutive size of the fornix and associated reactive gliosis. (**D**) Transverse brainstem section at the level of the pons, stained with LFB/H&E. Note the small size of the pons, which is estimated to be less than half its normal volume. The superior cerebellar peduncles are relatively normal, as is the tegmentum, but the size of the medial lemniscus and corticospinal tracts are somewhat reduced, albeit less than the pontine grey matter.

Post-mortem neuropathological examination (Patient 27.1) demonstrated callosal agenesis, hypoplasia of the pons, and corticospinal tracts as the most striking CNS abnormalities (Fig. 5). Additional findings included small brain size and coarse gyration pattern, hypoplastic hippocampi, and a small pons with markedly hypoplastic corticospinal bundles. Bodian silver stains showed a reduced number of axons within the hippocampus and other structures (data not shown). A major histopathological finding on general post-mortem examination was the presence of storage material in cardiomyocytes, Purkinje fibres, skeletal myocytes, bronchial chondrocytes, enteric smooth muscle cells, and hepatocytes; this material stained with periodic acid Schiff (PAS) (resistant to diastase digestion) on histological sections, indicating a carbohydrate or glycoprotein component. Electron microscopy demonstrated membrane-bound autophagic vacuoles containing phospholipid membranes, organelles, in particular mitochondria, and granular material consistent with glycogen as well as an excess of free glycogen. Neuropathological and general pathological findings on post-mortem were similar to those previously reported in two of our patients (Rogers *et al.*, 2011; Miyata *et al.*, 2014).

### Conditional *epg5* knock-down in *Drosophila*

**Figure 6:**
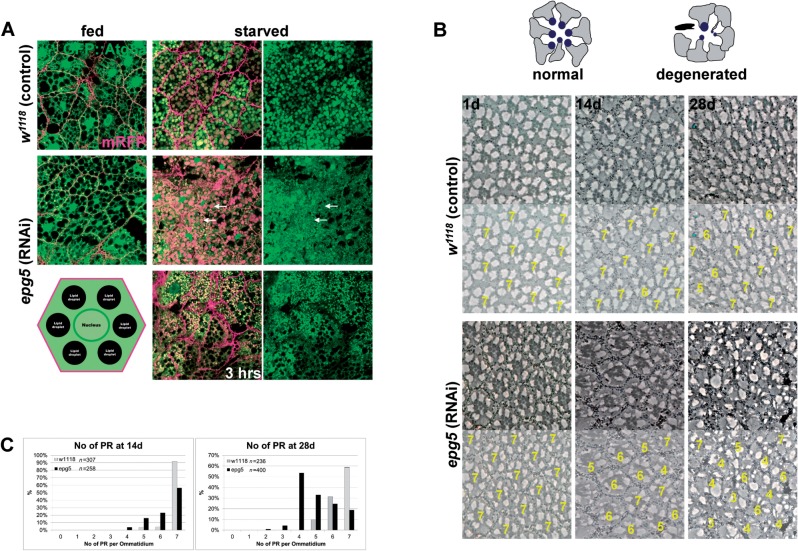
**Knock-down of *epg5* in *Drosophila*.** (**A**) Single confocal sections of fat bodies from fed and starved (6 h, unless otherwise indicated) larvae, either control or bearing RNAi-mediated downregulation of *epg5*. Red is membrane-bound RFP to highlight the cell membranes, green is the autophagy marker GFP::Atg8a. Arrows point to large autolysosomes from which GFP::Atg8a fluorescence is quenched on the inside, but is retained outside. Bottom left is a schematic drawing of an undigested fat body cell. (**B**) Semi-thin tangential eye sections from either control flies or flies bearing RNAi-mediated downregulation of *epg5* and aged 1, 14, and 28 days at 29°C to induce *epg5* downregulation. The drawing on top schematizes the process of cell degeneration and loss in this tissue. Below each section is an exemplary quantification of the photoreceptors in the ommatidia shown. (**C**) Graphs displaying the quantification of the number of photoreceptors (PR) in the ommatidia of the flies aged 14 and 28 days. Knock-down of *epg5* reduces the ommatidia with a normal number of photoreceptor neurons (7) and this phenotype increases with time (χ^2^ values are 166.5 and 721.6 with *P* < 0.0001 for 6 degrees of freedom).

We analysed the progression of autophagy in the *Drosophila* larval fat bodies, the equivalent of liver tissue in the fly, which is fully digested by autophagy during late larval and pupal stages. Upon starvation for 6 h, large autophagosomes and autolysosomes marked by GFP::Atg8a are readily observed in control wild-type larvae (Fig. 6A) and cellular structures, including nuclei, are not detectable as they have been degraded. However, when *epg5* (CG14299) is downregulated via the use of the collagen promoter and the Gal4/UAS expression system (CG14299-Gal4), large abnormal vacuoles, in which the GFP signal is quenched, accumulate (Fig. 6A). The shape of cellular structures, including the nuclei is preserved, indicating a failure in autophagic digestion. Analysis at an earlier time point reveals that autophagosomes initially form correctly as bright GFP::Atg8a punctae (Fig. 6A), which then stall and degenerate later on. This is consistent with defects in the late steps of autophagy after correct initiation and with a block in digestion of autolysosomes, as previously reported in EPG5-deficient human cells (Cullup *et al.*, 2013). Conditional downregulation of *epg5* specifically in the adult *Drosophila* retina (using GMR-Gal4 and a temperature-sensitive Gal80) reveals that, despite an intact retinal structure at Day 1 of adult life, loss of neurons and of retina structure evolves and is detected after 14 days and 28 days (Fig. 6B). Quantification of the number of remaining photoreceptor neurons in each ommatidium reveals a significant and incremental loss of cells over time (Fig. 6C). These data establish that the isolated loss of EPG5 function causes progressive neuronal degeneration in *Drosophila*, mirroring the situation in humans.

## Discussion

This is the first study to extensively describe genotype–phenotype correlations in a large cohort of patients with *EPG5*-related Vici syndrome, establishing the condition as a paradigm of neurodevelopmental conditions due to primary autophagy defects, and a probably not uncommon but under-recognized multisystem disorder.

*EPG5* mutations in our series were found throughout a wide range of different ethnicities. The vast majority of *EPG5* mutations were private, precluding future targeted screening of the large *EPG5* gene in patients presenting with suggestive clinical features. We identified only three recurrent *EPG5* mutations, in families with (possible or confirmed) Ashkenazi (p.Gln336Arg; *n* = 4), Maltese-Italian (p.Met2242CysfsX5; *n* = 2), and Caucasian or Middle-Eastern ancestry (p.Arg417X; *n* = 2), suggesting the presence of possible founder effects in some populations. Most *EPG5* mutations identified were single nucleotide substitutions with a predicted truncating effect on the EPG5 protein (Table 1 and Fig. 1). Identification of only one single pathogenic *EPG5* mutation in Patient 16.1 suggests the possibility of (compound) heterozygosity or homozygosity for non-overlapping genomic deletions in the large *EPG5* gene and emphasizes the need to complement current diagnostic strategies with techniques suitable to identify pathogenic copy number variations in future. Locus heterogeneity is another but less likely possibility, considering that >90% of patients presenting with the key diagnostic features were also found to have biallelic *EPG5* mutations.

Detailed review of the family histories in the 22 families remaining under follow-up (Supplementary Data) revealed a large number of cases of cancer (in particular breast, stomach, and skin) and, less frequently, Parkinson’s disease in relatives of patients with *EPG5*-related Vici syndrome. These observations are far too limited to draw any conclusions, especially as the incidence of cancer is increasing globally and also varies within different ethnic groups, but should prompt additional systematic prospective studies, to obtain more robust data concerning a potential association between the *EPG5* carrier state, and neoplastic and neurodegenerative disorders, respectively. Of note in this context, *EPG5* (known as *KIAA1632* at the time) was initially found to be mutated in breast cancer (Halama *et al.*, 2007), and there is a precedent of an increased frequency of Parkinson’s disease in families with lysosomal storage disorders (for review, see Brockmann and Berg, 2014). Moreover, an association of an increased cancer frequency and Parkinson’s disease has also recently been reported (Feng *et al.*, 2015), further supporting a link between tumorigenesis and neurodegenerative disorders linked in similar intracellular quality control pathways. The disproportionately high rate of (both monozygotic and dizygotic) twin pregnancies was another interesting but unexplained observation in our series.

Despite some variability of additional multi-systemic involvement (Supplementary Data), there was considerable homogeneity concerning a set of core clinical features (Table 2), probably reflecting that most *EPG5* mutations are predicted to cause at least partial loss of the EPG5 protein rather than more subtle alterations of functionally critical domains. In addition to the five features that have previously been considered to be diagnostic (McClelland *et al.*, 2010), we identified three additional features that are equally consistent in *EPG5*-related Vici syndrome and will help clinicians to make more informed decisions regarding genetic testing in suspected cases. The eight diagnostic features (Table 2) are highly predictive of *EPG5* involvement, with pathogenic *EPG5* mutations identified in >90% of cases where six or more of these features are present; however, some of these features (in particular cataracts, cardiomyopathy and immunodeficiency) may only evolve over time and are not necessarily present from birth.

We also obtained natural history data that will be useful for future clinical trials, indicating *EPG5*-related Vici syndrome as a severe disorder with a markedly reduced life expectancy. Although more detailed genotype–phenotype studies were hampered by the private nature of the majority of the *EPG5* mutations identified, tentative links between the class of mutation and the observed phenotype could be established. Whilst, not unexpectedly, most truncating *EPG5* mutations were associated with a severe phenotype, the only recurrent missense mutation in our series, p.Gln336Arg, was associated with a reduced likelihood of cataracts or a cardiomyopathy, and a relatively longer life expectancy. In addition, we demonstrated that patients with compound heterozygous *EPG5* mutations had longer survival times than those with homozygous *EPG5* mutations. The basis for this observation is currently unclear but may reflect over-representation of truncating mutations in homozygous compared to heterozygous patients.

A particular emphasis of this study was on a detailed characterization of neurological, neuroradiological, and neuropathological aspects. *EPG5*-related Vici syndrome emerged as a profoundly severe neurodevelopmental disorder, and our data suggest that acquisition of certain developmental skills virtually excludes *EPG5* involvement even if some of the other features are present. The finding of often severe epilepsy from infancy in two-thirds of patients puts *EPG5* within the rapidly expanding group of genes associated with early-onset epileptic encephalopathies, and in the context of other conditions, for example Lafora body disease (Ebrahimi-Fakhari *et al.*, 2014) or mTOR-related disorders (Lipton and Sahin, 2014), where severe epilepsy and dysregulation of the autophagy pathway have been associated. Our data also indicate that the natural history parallels that of a (neuro)degenerative condition: in those children surviving beyond infancy, there was progressive loss of skills and profound acquired microcephaly, suggesting that *EPG5*-related Vici syndrome is as much a neurodegenerative as a neurodevelopmental disorder. Intriguingly, neurodegeneration with mainly parkinsonian findings is also a feature of Chediak-Higashi (Silveira-Moriyama *et al.*, 2004) and Marinesco-Sjoegren syndromes (Byrne *et al.*, 2015), neurodevelopmental disorders with overlapping clinical features and linked in related molecular pathways (see below).

Agenesis of the corpus callosum, one of the original five diagnostic features, was found in all patients on systematic review of brain MRI findings (Fig. 3 and Supplementary Data) but is not specific *per se* and can often be syndromic (McClelland *et al.*, 2010); however, the isolated combination of callosal agenesis and pontine hypoplasia without other structural abnormalities is fairly distinct. Callosal agenesis and pontine hypoplasia has been reported in the ‘tubulinopathies’ due to mutations in the neuron-specific α- and β-tubulin genes (Cushion *et al.*, 2013), however, additional neuroradiological features (including basal ganglia abnormalities, cerebellar hypoplasia, and complex cortical malformations) commonly found in these disorders were not typically seen in *EPG5*-related Vici syndrome. Although pontine hypoplasia was prominent in our series, cerebellar abnormalities were not as marked as has been reported in pontocerebellar hypoplasia (Uhl *et al.*, 1998), or *SNX14-*associated cerebellar ataxia and intellectual disability (Thomas *et al.*, 2014; Akizu *et al.*, 2015). Like *EPG5*, *SNX14* has a putative role in endosome/autophagosome interaction with lysosomes, but it is currently uncertain how these functionally similar proteins cause overlapping, but nevertheless distinct CNS malformations.

In addition to non-specific features such as delayed myelination, an interesting and previously unreported finding was the presence of abnormal low T_2_ signal in the thalamus in five patients, apparently evolving over time. Similar thalamic changes have been previously reported as a relatively specific feature of lysosomal storage disorders (Autti *et al.*, 2007). Taken together with overlapping clinical and histopathological features, these findings provide further evidence that primary autophagy disorders, such as Vici syndrome and primary lysosomal storage disorders, are intricately linked in the same pathogenic mechanisms, as has been suggested previously (Settembre *et al.*, 2008).

Systematic analysis of muscle biopsy findings (Fig. 4 and Supplementary Data) confirmed a skeletal myopathy, previously documented in more detail in only two cases (Al-Owain *et al.*, 2010; McClelland *et al.*, 2010), as a consistently associated finding. Whilst numerous vacuoles on both light and electron microscopy are the most ominous finding and define the *EPG5*-related skeletal muscle myopathy as a primary vacuolar myopathy, vacuoles can be absent or inconspicuous and other features may be equally or more prominent, including marked variability in fibre size with type 1 predominance and atrophy (fibre type disproportion), increase in internal and centralized nuclei, and increased glycogen storage. In few cases we also observed focal areas of sarcomeric disorganization, resembling minicores, suggesting an overlap with the core myopathies (Jungbluth *et al.*, 2011). Respiratory chain enzyme abnormalities were another novel observation in four patients, supporting secondary mitochondrial dysfunction, already suggested by abnormalities of mitochondrial internal structure and positioning reported in earlier studies (Cullup *et al.*, 2013), as a possible downstream effect of defective autophagy. Based on the observations above, histopathological features in *EPG5*-related Vici syndrome may mimic a number of primary neuromuscular disorders, in particular the vacuolar myopathies (Malicdan and Nishino, 2012) and centronuclear myopathies (Jungbluth *et al.*, 2008), conditions that, interestingly, have been linked with primary and secondary defects of the autophagy pathway (Jungbluth and Gautel, 2014). The defects implicated in Danon disease (Nishino *et al.*, 2000) and X-linked myopathy with excessive autophagy (MEAX) (Ramachandran *et al.*, 2013), in particular impaired autolysosomal fusion and defective intralysosomal digestion, affect the same part of the autophagy pathway also affected in EPG5 deficiency. Considering common features of increased glycogen storage and mitochondrial abnormalities, *EPG5*-related Vici syndrome also ought to be considered in genetically unresolved ‘glycogen storage’ or ‘mitochondrial’ disorders suspected on histopathological grounds.

The differential diagnosis of *EPG5*-related Vici syndrome includes a number of syndromes with overlapping clinical features. Marinesco-Sjoegren syndrome and related disorders share cataracts and a skeletal myopathy with or without sensorineural deafness; however, acquired microcephaly and profound failure to thrive are not common features and the overall degree of developmental delay is usually less severe (Krieger *et al.*, 2013). Hypopigmentation and immune defects are also features of Chediak-Higashi syndrome and related primary immunodeficiency syndromes. Chediak-Higashi syndrome also features neuro-ophthalmological similar to those seen in *EPG5*-related Vici syndrome (Filloux *et al.*, 2014), indicating that both conditions belong to the same group of syndromic albinism.

The multitude of symptoms associated with *EPG5*-related Vici syndrome reported in the present study implicates the autophagy pathway in the normal formation and functioning of a wide range of organ systems (brain, eye, hearing, heart, lung, liver, kidney, immune system, blood) and, by proxy, organ-specific disease. While in some of these organs a role of the autophagy pathway is already recognized, in others such a role has not yet been considered. A striking and previously unrecognized feature in our series was profound failure to thrive despite adequate calorie intake, with almost all patients dropping below the 0.4th centile for weight within the first year of life. Low weight, probably due to reduced adipose tissue or reduced muscle mass, is also a feature in *Epg5* knockout mice (Zhao *et al.*, 2013*a*), suggesting a specific association with EPG5 deficiency.

Our observation of a common occurrence of structural and degenerative changes affecting the same organ in the same patient suggests that *EPG5*-related Vici syndrome is as much a disorder of embryonic organ development as of normal organ function. For example, a substantial proportion of patients in our series had structural congenital cardiac defects and developed a severe cardiomyopathy later in life (Supplementary Data), probably reflecting rapid accumulation of vacuoles and abnormal material in metabolically highly active cardiomyocytes, as demonstrated on post-mortem myocardial examination in one family (Rogers *et al.*, 2011); increased oxidative stress due to accumulation of damaged mitochondria is possibly a main contributory component.

The dual effects of defective autophagy on cellular development and maintenance in *EPG5*-related Vici syndrome are particularly pertinent on the level of the CNS, where the present study provides evidence for a neurodegenerative component evolving in the context of a neurodevelopmental defect, also supported by findings of a neuropathological phenotype resembling human amyotrophic lateral sclerosis in the *Epg5* knockout mouse (Zhao *et al.*, 2013*a*). A general role of autophagy in neuronal embryogenesis is supported by the finding of severe neural tube defects in the *Ambra1* knockout mouse but remains to be further elucidated (Cecconi *et al.*, 2007; Fimia *et al.*, 2007). Moreover, certain stem cells involved in neurodevelopment may also have a role in neurodegeneration, suggesting that defects in the pathways leading to aberrant development may also result in later neurodegeneration, due to a pleiotropic effect of some neurodevelopmental genes on the nervous system (Zhang *et al.*, 2013). These observations place *EPG5*-related Vici syndrome within an emerging group of early-onset neurodevelopmental and neurodegenerative disorders associated with defective autophagy (reviewed in Ebrahimi-Fakhari *et al.*, 2014, 2015) such as static encephalopathy in childhood with neurodegeneration in adulthood (SENDA) due to recessive mutations in *WDR45* (Haack *et al.*, 2012), and a recently recognized syndromic form of early-onset ataxia due to recessive mutations in *SNX14* (Thomas *et al.*, 2014; Akizu *et al.*, 2015).

To explore the effects of EPG5 deficiency on neuronal maintenance, we generated a conditional neuronal *Drosophila* knock-down (Fig. 6). First we confirmed that in *Drosophila* loss of *epg5* function leads to defects in the autophagic process of digestion of the larval fat bodies triggered by starvation, which essentially recapitulates the defects in late events in the autophagy cycle reported in human cells (Cullup *et al.*, 2013) and validates the use of *Drosophila* as a model to study EPG5 function. *Drosophila* is widely used for the study of neurodegenerative conditions, including those resulting from autophagy alterations. These neurodegenerative phenotypes are often studied in the eye photoreceptor neurons, as this is the most accessible and life-dispensable part of the fly nervous system (Nisoli *et al.*, 2010; Napoletano *et al.*, 2011). In this system we were able to demonstrate the requirement for *epg5* for preservation of neuronal cells during the fly lifespan, suggesting that the progressive loss of neuronal cells observed in the fly may be the basis for some of the neurological features seen in EPG5-deficient humans.

## Conclusion

Consistent clinical, neuroradiological, and neuropathological findings suggest *EPG5*-related Vici syndrome as a paradigm of a new class of multisystem disorders featuring substantial overlap with other multisystem presentations, in particular those due to primary glycosylation defects, mitochondrial disease, and lysosomal and glycogen storage disorders. A number of similar conditions with overlapping features have been described in association with defects concerning intracellular trafficking and lysosomal transport, suggesting that disorders intricately linked in the same pathways (in particular those concerning autophagosome and lysosome biology) share a recognizable clinical signature (Ebrahimi-Fakhari *et al.*, 2015). Moreover, we postulate that *EPG5*-related Vici syndrome is as much a disorder of embryonic organ development as of degeneration. Evolution of neurodegenerative features over time indicates an intriguing link between neurodevelopment and neurodegeneration, also supported by features of neurodegeneration in *epg5*-deficient *D. melanogaster*, and recent implication of other autophagy regulators in late-onset neurodegenerative disease.

## Supplementary Material

Supplementary Data

Supplementary material
