# Supplementary material for: EPG5-related Vici syndrome: a paradigm of neurodevelopmental disorders with defective autophagy
Source: Brain. 2016 Feb 17;139(3):765–81. doi: 10.1093/brain/awv393 (PMC4766378; doi:10.1093/brain/awv393)
Supplement: Supplementary Data [file awv393_supplementary_data.zip › brain-2015-01466-File013.pdf]

| Family | Origin | Ethnicity | Consanguinity | Vici history | Cancer history | Neurodegenerative history | Other family history |
|---|---|---|---|---|---|---|---|
| 1 | Italy | Caucasian | No | 2 siblings | - | - | - |
| 2 | Italy | Caucasian | No | No | - | - | Vitiligo |
| 3 | UK | British-Asian | No | No | Gastric | Parkinson disease, dementia x2 | Vitiligo |
| 4 | Germany | Turkish | Yes | 2 siblings | - | - | - |
| 5 | Netherlands | Turkish | Yes | Large consanguineous family where many children died young | - | - | - |
| 6 | USA | Caucasian | No | No | - | - | - |
| 7 | USA | Caucasian | Yes | No | - | - | - |
| 8 | USA | Caucasian | No | 2 siblings affected | Leukaemia x2, colon, breast | Parkinson disease | Two sets of twins on maternal side of family |
| 9 | Saudi Arabia | Arabic | Yes | 3 siblings affected | No | No | Large number of relatives with fairer hair than expected for ethnic origin |
| 10 | Japan | Japanese | No | 2 siblings affected | No | No | No |
| 11 | Malta | Caucasian | No | No | No | No | No |
| 12 | USA | Caucasian | No | No | Gastric | Epilepsy | Two sets of twins on maternal side of family |
| 13 | UAE | Arabic | Yes | No | No | No | No |
| 14 | Egypt | Arabic | Yes | 4 siblings affected | No | No | No |
| 15 | Israel | Israeli-Arabic | Yes | 3 siblings, 1 cousin | - | - | - |
| 16 | UK | Caucasian | No | No | Breast, melanoma x2 | No | No |
| 17 | Germany | Turkish | Yes | 3 siblings (including 1 set of twins) affected, 2 uncles also affected | - | - | - |
| 18 | Australia/ Greece | Caucasian | Yes | 2 siblings, 1 cousin affected | Gastric, melanoma x2, lung, liver | - | - |
| 19 | UAE | Arabic | Yes | No | Breast | No | No |
| 20 | Italy | Caucasian | No | No | No | No | No |
| 21 | Oman | Arabic | Yes | 2 siblings affected | No | No | No |
| 22 | Saudi Arabia | Arabic | Yes | No | No | No | 2 siblings with Gaucher's disease |
| 23 | Germany | Caucasian | No | 2 siblings affected | Gastric, colon | No | Cataracts in 1st degree relative with no other features of Vici syndrome |
| 24 | USA | Caucasian | No | 2 siblings affected | No | No | Second degree relative has Charcot Marie-Tooth disease |
| 25 | Brazil | Caucasian | No | 2 siblings affected | No | No | No |
| 26 | Israel | Ashkenazi | Yes | Non-identical twin siblings; one affected | No | No | No |
| 27 | USA | Caucasian | No | No | No | No | No |
| 28 | Israel | Israeli-Arabic | Yes | Yes, cousin affected | No | No | No |
| 29 | USA | Ashkenazi | No | No | Colon and pancreatic on paternal side, pancreatic and sarcoma on maternal side | No | No |
| 30 | USA | Caucasian | No | No | No | No | No |

**Supplemental table 1**
