# Supplementary material for: EPG5-related Vici syndrome: a paradigm of neurodevelopmental disorders with defective autophagy
Source: Brain. 2016 Feb 17;139(3):765–81. doi: 10.1093/brain/awv393 (PMC4766378; doi:10.1093/brain/awv393)
Supplement: Supplementary Data [file awv393_supplementary_data.zip › brain-2015-01466-File014.pdf]

Patient	Diagnosis	Details	Corpus callosum	Cataract	Skin	Cardio-myopathy	Immune	Neonatal presentation	Profound delay development	Failure to thrive	Microcephaly
1.1	Vici	EPG5 +, clinical phenotype	Absent	Yes	Pallor	-	Yes	Yes	Yes	Yes	Yes
1.2	Vici	EPG5 +, clinical phenotype	Absent	Yes	Pallor	-	Yes	Yes	Yes	Yes	Yes
2.1	Vici	EPG5 +, clinical phenotype	Absent	Yes	Pallor	Yes	Yes	Yes	Yes	Yes	Yes
3.1	Vici	EPG5 +, clinical phenotype	Absent	Yes	Pallor	Yes	Yes	Yes	Yes	Yes	Yes
4.1	Vici	EPG5 +, clinical phenotype	Absent	Yes	Pallor	Yes	Yes	Yes	Yes	Yes	Yes
5.1	Vici	EPG5 +, clinical phenotype	Absent	No	Pallor	Yes	Yes	Yes	Yes	Yes	-
5.2	Vici	EPG5 +, clinical phenotype	Absent	Yes	Pallor	Yes	Yes	Yes	Yes	Yes	-
6.1	Vici	EPG5 +, clinical phenotype	Absent	Yes	Pallor	Yes	Yes	Yes	Yes	Yes	No
7.1*	Vici	EPG5 +, clinical phenotype	Absent	Yes	Pallor	Yes	Yes	Yes	Yes	Yes[4]	Yes
8.1	Vici	EPG5 +, clinical phenotype	Absent	Yes	Pallor	Yes	Yes	Yes	Yes	Yes[4]	Yes
8.2	Vici	EPG5 +, clinical phenotype	Absent	Yes	Pallor	Yes	Yes	Yes	Yes	Yes	Yes
9.1	Vici	EPG5 +, clinical phenotype	Absent	Yes	Pallor	Yes	Yes	Yes	Yes	Yes	Yes
10.1	Vici	EPG5 +, clinical phenotype	Absent	Yes[2]	Pallor	Yes	Yes	Yes	Yes	Yes	Yes
10.2	Presumed Vici	Clinical phenotype, EPG5 mutation confirmed in relative	Absent	Yes[2]	Pallor	Yes	Yes	Yes	Yes	Yes	-
11.1	Vici	EPG5 +, clinical phenotype	Absent	Yes	Pallor	Yes	Yes	No[3]	Yes	Yes	Yes
12.1	Vici	EPG5 +, clinical phenotype	Absent	Yes	Pallor	Yes	Yes	Yes	Yes	Yes	Yes
13.1	Vici	EPG5 +, clinical phenotype	Absent	Yes	Pallor	Yes	Yes	Yes	Yes	Yes	Yes
14.1	Vici	EPG5 +, clinical phenotype	Absent	Yes[2]	Pallor	Yes	Yes	Yes	Yes	Yes	Yes
14.2	Presumed Vici	Clinical phenotype, EPG5 mutation in relative	-	-	Pallor	-	Yes	Yes	Yes	-	-
14.3	Presumed Vici	Clinical phenotype, EPG5 mutation in relative	-	Yes	Pallor	Yes	Yes	Yes	Yes	Yes	-
14.4	Presumed Vici	Clinical phenotype, EPG5 mutation in relative	Absent	Yes	Pallor	-	Yes	Yes	Yes	-	-
15.1	Vici	EPG5 +, clinical phenotype	Absent	Yes	Pallor	Yes	Yes	Yes	Yes	-	-
15.2	Vici	EPG5 +, clinical phenotype	Absent	Yes	Pallor	Yes	Yes	Yes	Yes	-	-
15.3	Presumed Vici	Clinical phenotype, EPG5 mutation in	Absent	Yes	Pallor	Yes	Yes	Yes	Yes	-	-

		relative									
15.4	Presumed Vici	Clinical phenotype, EPG5 mutation in relative	Absent	Yes	Pallor	Yes	Yes	Yes	Yes	-	-
16.1*	Vici	>EPG5 +, clinical phenotype	Absent	No	Pallor	No	Yes	Yes	Yes	-	Yes
17.1	Vici	EPG5 +, clinical phenotype	Absent	No	Pallor	Yes	Yes	Yes	Yes	Yes	Yes
17.2	Presumed Vici	Clinical phenotype, EPG5 mutation in relative	Absent	No	Pallor	Yes	Yes	Yes	Yes	Yes	Yes
17.3	Presumed Vici	Clinical phenotype, EPG5 mutation in relative	Absent	-	Pallor	-	-	-	Yes	-	-
17.4	Presumed Vici	Clinical phenotype, EPG5 mutation in relative	Absent	-	Pallor	-	-	-	Yes	-	-
17.5	Vici	EPG5 +, clinical phenotype	Absent	No	Pallor	Yes	Yes	Yes	Yes	Yes	Yes
18.1	Vici	EPG5 +, clinical phenotype	Absent	Yes	Pallor	Yes	Yes	Yes	Yes	Yes	No
18.2	Presumed Vici	Clinical phenotype, EPG5 mutation in relative	Absent	Yes	Pallor	Yes	Yes	Yes	Yes	Yes	-
18.3	Presumed Vici	Clinical phenotype, EPG5 mutation in relative	Absent	-	Pallor	No	Yes	Yes	Yes	Yes	-
19.1	Vici	EPG5 +, clinical phenotype	Absent	Yes	Pallor	Yes	Yes	No[3]	Yes	Yes	Yes
20.1	Vici	EPG5 +, clinical phenotype	Absent	No	Pallor	Yes	Yes	Yes	Yes	Yes	Yes
21.1	Vici	EPG5 +, clinical phenotype	Absent	Yes	Pallor	Yes	Yes	Yes	Yes	Yes	Yes
22.1	Vici	EPG5 +, clinical phenotype	Absent	Yes	Normal skin	-	Yes	Yes	Yes	Yes	Yes
23.1	Vici	EPG5 +, clinical phenotype	Absent	Yes	Pallor	No	Yes	Yes	Yes	Yes[4]	Yes
23.2	Vici	EPG5 +, clinical phenotype	Absent[1]	Yes	Pallor	-	Yes	Yes	-	-	-
24.1	Vici	EPG5 +, clinical phenotype	Absent	No	Pallor	No	Yes	No[3]	Yes	Yes	Yes
24.2	Vici	EPG5 +, clinical phenotype	Absent	Yes	Pallor	No	Yes	No[1]	Yes	Yes	No
25.1	Vici	EPG5 +, clinical phenotype	Absent	Yes	Pallor	No	Yes	Yes	Yes	Yes	Yes
25.2	Presumed Vici	Clinical phenotype, EPG5 mutation in relative	Absent	No	Pallor	-	No	Yes	-	-	-
26.1*	Vici	EPG5 +, clinical phenotype	Absent	No	Pallor	No	Yes	Yes	Yes	Yes	Yes
27.1	Vici	EPG5 +, clinical phenotype	Absent	Yes	Pallor	Yes	Yes	Yes	Yes	Yes	No
28.1	Vici	EPG5 +, clinical phenotype	Absent	No	Pallor	Yes	Yes	Yes	Yes	Yes	Yes
28.2	Vici	EPG5 +,	Absent	Yes	Pallor	Yes	Yes	Yes	Yes	Yes	Yes

		clinical phenotype									
29.1*	Vici	EPG5 +, clinical phenotype	Absent	Yes	No	Transient	Yes **	Yes	Yes	No[4]	Yes
30.1	Vici	EPG5 +, clinical phenotype	Absent	Yes	Pallor	Yes	Yes	No[3]	Yes	Yes[4]	Yes

Supplemental table 2
