# Supplementary material for: EPG5-related Vici syndrome: a paradigm of neurodevelopmental disorders with defective autophagy
Source: Brain. 2016 Feb 17;139(3):765–81. doi: 10.1093/brain/awv393 (PMC4766378; doi:10.1093/brain/awv393)
Supplement: Supplementary Data [file awv393_supplementary_data.zip › brain-2015-01466-File015.pdf]

Patient	Diagnosis	Cardiomyopathy	Details and additional findings
1.1	Vici	NA	NA
1.2	Vici	NA	NA
2.1	Vici	Yes	Progressive systolic dysfunction with LV dilatation and mitral regurgitation (US)
3.1	Vici	Yes	Hypertrophic cardiomyopathy (US)
4.1	Vici	Yes	Hypertrophic cardiomyopathy; PFO (US)
5.1	Vici	Yes	Hypertrophic cardiomyopathy (US)
5.2	Vici	Yes	Hypertrophic cardiomyopathy (US)
6.1	Vici	Yes	Dilated cardiomyopathy (US)
7.1	Vici	Yes	Dilated cardiomyopathy (US)
8.1	Vici	Yes	Dilated cardiomyopathy with LVH (US)
8.2	Vici	Yes	Dilated cardiomyopathy (US)
9.1	Vici	Yes	Hypertrophic cardiomyopathy with mild left atrial and ventricular enlargement (US)
10.1	Vici	Yes	Dilated cardiomyopathy (US)
10.2	PV	Yes	No further details available
11.1	Vici	Yes	Dilated cardiomyopathy (US)
12.1	Vici	Yes	Transient cardiomyopathy with residual mild dilated cardiomyopathy (US)
13.1	Vici	Yes	Hypertrophic cardiomyopathy with evidence of biventricular hypertrophy (ECG) and mild posterior wall thickness (US)
14.1	Vici	Yes	Dilated cardiomyopathy with LVH (US)
14.2	PV	NA	NA
14.3	PV	Yes	No further details available
14.4	PV	NA	NA
15.1	Vici	Yes	Hypertrophic cardiomyopathy (US)
15.2	Vici	Yes	Hypertrophic cardiomyopathy (US)
15.3	PV	Yes	Hypertrophic cardiomyopathy (US)

15.4	PV	Yes	Hypertrophic cardiomyopathy (US)
16.1	Vici	No	Normal cardiac US
17.1	Vici	Yes	Hypertrophic cardiomyopathy, progressive (US)
17.2	PV	Yes	Thickened myocardium (US)
17.3	PV	NA	NA
17.4	PV	NA	NA
17.5	Vici	Yes	Cardiomyopathy, not further specified (US)
18.1	Vici	Yes	Hypertrophic cardiomyopathy, with mild LV and moderate RV hypertrophy (US); hypoplastic aortic arch
18.2	PV	Yes	Cardiomyopathy, not further specified (detected on post mortem study)
18.3	PV	No	PFO (US)
19.1	Vici	Yes	Dilated cardiomyopathy with LVH and small right ventricle (US); multiple atrial septal defects
20.1	Vici	Yes	Dilated cardiomyopathy with mitral insufficiency (US)
21.1	Vici	Yes	Hypertrophic cardiomyopathy with dilated left ventricle (US)
22.1	Vici	NA	Cardiac US not performed, no clinical evidence of cardiac failure
23.1	Vici	No	Mild septal and RVH in neonatal period (US) but resolved; PFO
23.2	Vici	NA	NA
24.1	Vici	No	Essentially normal cardiac function with minor mitral and tricuspid regurgitation (US)
24.2	Vici	No	Normal cardiac US
25.1	Vici	No	Mitral insufficiency, no cardiomyopathy (US)
25.2	PV	NA	NA
26.1	Vici	NA	Cardiac US not performed, no clinical evidence of cardiac failure
27.1	Vici	Yes	Dilated cardiomyopathy with marked ventricular dilatation (US)
28.1	Vici	Yes	Dilated cardiomyopathy with some hypertrophy (US)
28.2	Vici	Yes	Concentric hypertrophy of left ventricle with normal function (US)
29.1	Vici	NA	Cardiac US not performed, no clinical evidence of cardiac failure
30.1	Vici	Yes	Mild LVH, systolic heart failure (US)

Supplemental table 3
