# Supplementary material for: EPG5-related Vici syndrome: a paradigm of neurodevelopmental disorders with defective autophagy
Source: Brain. 2016 Feb 17;139(3):765–81. doi: 10.1093/brain/awv393 (PMC4766378; doi:10.1093/brain/awv393)
Supplement: Supplementary Data [file awv393_supplementary_data.zip › brain-2015-01466-File016.pdf]

**Neuroradiological features**	**Prevalence in study population (n = 18)**
**Key features**	
Complete Agenesis of Corpus Callosum	n = 18 (100 %)
Pontine Hypoplasia	n = 18 (100 %)
Delayed myelination	n = 18 (100 %)
Reduction in white matter bulk	n = 18 (100 %)
Underopercularisation of Sylvian fissures	n = 18 (100 %)
**Less common features**	
Low T2 signal in thalamus	n= 5 (31%) (High T1 signal seen in one of the 5 cases)
Simplified sulcation	n = 2 (13%)
Cerebellar abnormalities	n = 3 (mild deficiency of inferior vermis) (19 %)

Supplemental Table 4
