# Supplementary material for: EPG5-related Vici syndrome: a paradigm of neurodevelopmental disorders with defective autophagy
Source: Brain. 2016 Feb 17;139(3):765–81. doi: 10.1093/brain/awv393 (PMC4766378; doi:10.1093/brain/awv393)
Supplement: Supplementary Data [file awv393_supplementary_data.zip › brain-2015-01466-File017.pdf]

Pat	IFSV	I/CN	Vac	Gly	T1H/CFTD	T1P	Cor	RCE	EM
2.1	+	+	+	-	+	-	-	ND	Vacuoles; abnormal mitochondria
3.1	+	+	+	+	+	+	-	ND	Central nuclei; vacuoles; abnormal mitochondria
4.1	+	+	+	+	+	-	-	ND	Central nuclei; vacuoles; abnormal mitochondria
6.1	+	+	+	-	+	+	-	ND	Mild excess of lipofuscin for age; no vacuoles
7.1	+	-	-	-	+	+	+	ND	Vacuoles in rare fiber; rare minicores
9.1	+	+	+	+	+	+	-	ND	Subsarcolemmal vacuoles, glycogen and mitochondria
11.1	+	+	-	+	+	-	-	Normal	ND
12.1	+	-	-	-	+	+	-	Normal	ND
16.1	+	+	+	+	-	-	-	Normal	Subsarcolemmal accumulation of autophagic vacuoles, glycogen and mitochondria
18.1	+	+	+	-	-	-	-	Reduced complexes I, III and IV	Central nuclei; subsarolemmal autophagic vacuoles; increased glycogen; large mitochondria; some sarcomeric disorganisation
19.1	+	+	-	-	+	-	-	Reduced complex IV	ND
22.1	+	+	+	+	-	-	-	Normal	ND
24.1	+	+	+	-	+	-	-	ND	Increased glycogen; abnormally shaped and structured mitochondria; some sarcomeric disorganization
26.1	+	-	+	-	-	-	-	ND	ND
27.1	+	+	-	+	-	-	-	ND	Abundant debris, free and membrane bound glycogen
28.1	-	-	+	-	-	-	-	Normal	Autophagocytic vacuoles; increased glycogen storage; structurally abnormal mitochondria
29.1	+	-	+	-	-	-	-	Reduced complexes I, III and IV	Accumulation of subsarcolemmal inclusions; increase in glycogen; alterations of mitochondrial distribution, size and internal structure

Supplemental Table 5
