# Supplementary material for: EPG5-related Vici syndrome: a paradigm of neurodevelopmental disorders with defective autophagy
Source: Brain. 2016 Feb 17;139(3):765–81. doi: 10.1093/brain/awv393 (PMC4766378; doi:10.1093/brain/awv393)
Supplement: Supplementary Data [file awv393_supplementary_data.zip › brain-2015-01466-File018.pdf]

Department of Paediatric Neurology – Neuromuscular Service
Evelina Children's Hospital
Guy's & St Thomas' NHS Foundation Trust
Westminster Bridge Road
London SE1 7EH
United Kingdom25/10/201325/10/2013

Please return form to:
Dr Heinz Jungbluth
Children's Neuroscience Centre
F01 – Staircase D South Wing
St Thomas' Hospital
Westminster Bridge Road
London SE1 7EH
United Kingdom
Phone: 0044 2071883998
Fax: 0044 2071884629
e-mail: Heinz.Jungbluth@gstt.nhs.uk

Diagnostic service for autophagy-related disorders

Patient details

Diagnosis ____

Name _________________________ Date of Birth _____________________

Identifier (NHS number)_____________ Address ___________________________

____

Country ________________________ Alive/dead at age _________________

Patient 's country of origin/ethinicty_______________________________________

Name of parents and date of birth____

Clinician _________________________Hospital _____________________________

Clinician email ____

Gene loci

EPG5 ❑ *SIL1* ❑ *LAMP2* ❑ *VMA21* ❑

Please attach genetic result report if already available

Patient samples available

DNA	❑ Yes	❑ No	stored at ___________________________
Fibroblasts	❑ Yes	❑ No	stored at ___________________________
Muscle	❑ Yes	❑ No	stored at ___________________________
Others	❑ Yes	❑ No	specify ___________________________

Family history

Parental consanguinity ❑ Yes ❑ No If yes, give details ___________________

Other affected family members ❑ Yes ❑ No If yes, give details _____________

Family history ❑ Vitiligo ❑ Cancer ❑ Other If yes, give details _____________

____

Please attach a family pedigree

Perinatal details

Foetal movements ❑ Reduced ❑ Normal ❑ Increased

Amniotic fluid ❑ Normal ❑ Polyhydramnios ❑ Oligohydramnios

Gestation at birth _________________ Birth weight ________________________

Birth length ________________________ Birth head circumference _____________

Neonatal ❑ Hypotonia ❑ Reduced movements
❑ Feeding difficulties ❑ Tube feeding ❑ Hypoglycaemia
❑ Respiratory impairment ❑ Ventilation if yes, duration ________

Developmental history

Motor development ❑ Normal ❑ Delayed ❑ Unknown
Age first sat alone ____________________ Age first walked alone _________________
Best functional ability ❑ Sitting ❑ Walking ❑ Running

Speech development ❑ Normal ❑ Delayed ❑ Unknown

Hearing ❑ Normal ❑ Impaired ❑ Unknown

Vision ❑ Normal ❑ Impaired ❑ Unknown

CNS involvement

Microcephaly ❑ Yes ❑ No
Current head circumference ____________ Age at measurement __________

Learning difficulties ❑ No ❑ Mild ❑ Moderate ❑ Severe

Seizure ❑ Yes ❑ No

Type seizure ____

MRI brain

❑ Callosal agenesis ❑ Cerebellar atrophy ❑ Pontocerebellar hypoplasia

❑ Neuromigrational defects ❑ Other ______________________________________

Please attach MRI report

Muscle involvement

Weakness ❑ None ❑ Facial ❑ Extraocular

❑Axial ❑ Proximal ❑ Distal ❑ Generalized

Muscle biopsy ❑ Yes ❑ No ❑ Unknown

Age at muscle biopsy _________________ Biopsy site _________________

Pathological features: ❑ Fibre size variability ❑ Increased internal nuclei

❑ Vacuoles ❑ Type 1 fibre predominance

❑ Cores ❑ Abormal glycogen storage

❑ Others __________________________

Please attach muscle biopsy report

Nerve involvement

Deep tendon reflexes ❑ Normal ❑ Reduced ❑ Absent ❑ Brisk

EMG/NCS ❑ Normal ❑ Abnormal ❑ Not done/unknown

Nerve biopsy ❑ Normal ❑ Abnormal ❑ Not done/unknown

Please attach EMG/NCS report
Please attach nerve biopsy report

Skin involvement

❑ Normal skin ❑ Hypopigmentation ❑ Hyperpigmentation ❑ Vitiligo ❑ Ichthyosis

Immune involvement

❑Yes ❑ No ❑Unknown

❑ B cell defect ❑ T cell defect ❑ Combined defect

Frequent infections ❑ Yes ❑ No

If yes, provide details of infections

____

Haematological involvement

❑ Yes ❑ No ❑ Unknown

❑ Anaemia ❑ Leucopaenia ❑ Thrombocytopaenia

Please provide details ____

Ocular involvement

❑Yes ❑ No ❑ Unknown

❑ Cataracts ❑ Optic atrophy ❑ Retinal abnormalities

VEP/ERG ❑ Yes ❑ No

Please provide details ____

Auditory involvement

Hearing impairment ❑ Yes ❑ No ❑ Unknown

*If yes, please provide details (sensorineural ? conductive?)*______________________

BAER ❑ Yes ❑ No

Please provide details ____

Cardiac involvement

❑ Yes ❑ No ❑ Unknown

Cardiomyopathy ❑ No ❑ Dilated ❑ Hypertrophic

Please provide details/attach ultrasound report ___________________________

Pulmonary involvement

❑ Yes ❑ No ❑ Unknown

❑ Pulmonary hypoplasia ❑ frequent infections ❑ Unknown

Please provide details ____

Thyroid involvement

❑ Yes ❑ No ❑ Unknown

❑ Thyroid agenesis ❑ Hypothyroidism ❑ Hyperthyroidism

*Please provide details*____

Thymic involvement

❑ Yes ❑ No ❑ Unknown

Thymic aplasia ❑ Yes ❑ No ❑ Unknown

*Please provide details*____

Liver involvement

❑ Yes ❑ No ❑ Unknown

Hepatomegaly ❑ Yes ❑ No

Abnormal liver enzymes ❑ Yes ❑ No

*Please provide details*____

Renal involvement

❑ Yes ❑ No ❑ Unknown
❑ Hydonephrosis ❑ Renal tubular acidosis ❑ Electrolyte disturbance

Please provide details ____

Failure to thrive?

❑ Yes ❑ No ❑ Unknown

*Most recent weight and age at which weighed*____________________________

Other system involvement

Please give details of any other systemic features that you have noted in this patient, or any additional information that you would like to add:

____
____
____
____
____
____
____
____
____
____
____
____

Attached reports

If available (please tick as applicable) please provide the following information:

❑ Clinical letters ❑ Genetic result(s) ❑ Muscle biopsy report
❑ EMG/NCS report ❑ MRI brain report ❑ Cardiac ultrasound
❑ Family pedigree ❑ Immunology results ❑ CXR_______________
❑ Nerve biopsy report ❑ Abdominal US ❑ Other _____________

THANK YOU !
