# Supplementary material for: EPG5-related Vici syndrome: a paradigm of neurodevelopmental disorders with defective autophagy
Source: Brain. 2016 Feb 17;139(3):765–81. doi: 10.1093/brain/awv393 (PMC4766378; doi:10.1093/brain/awv393)
Supplement: Supplementary Data [file awv393_supplementary_data.zip › brain-2015-01466-File020.pdf]

SUPPLEMENTAL FILES

Supplemental File 1 (SF1)

Referral form for diagnostic *EPG5* screening

Supplemental Table 1 (ST1)

Family histories

Details of the family history in 22 families with *EPG5*-related Vici syndrome under active follow-up. - = no information available

Supplemental Table 2 (ST2)

Detailed clinical features in patients with *EPG5*-related Vici syndrome

Details of the common clinical features in 50 patients with Vici syndrome, 38 with confirmed *EPG5* mutations, and 12 who have not had *EPG5* testing but do have the clinical phenotype and the mutation confirmed in an affected relative. - = no information available; > only one mutation identified; * = recurrent pGly336Arg mutation; [1] = possibly hypoplastic corpus callous, cranial ultrasound examination only; [2] = cataracts suspected because of reduced light reflex; [3] = these infants presented outside the neonatal period, between the 2nd and 4th month of life. [4] = these patients had failure to thrive in infancy but following commencement of gastrostomy feeds had normal weight at 1 year (Patient 30.1), 2 years (Patient 23.1), 4 years (Patient 7.1), 8 years (Patient 8.1 and Patient 29.1). In most children, an underlying immune defect was suggested by the number and the type of recurrent infections, but not all had a formal immunological assessment.

Supplemental Table 3 (ST3)

Cardiological features in *EPG5*-related Vici syndrome

Cardiac features in 50 patients with (presumed) *EPG5*-related Vici syndrome. NA = information not available or insufficient; PFO = persistent foramen ovale; PV = presumed Vici syndrome (patients with clinical features of Vici syndrome and confirmation of causative

EPG5 mutations in a relative); LV = left ventricular; LVH = left ventricular hypertrophy; RV = right ventricular; RVH = right ventricular hypertrophy; US = ultrasound.

Supplemental Table 4 (ST4)

Neuroradiological features of *EPG5*-related Vici syndrome

Neuroradiological features in 18 patients with genetically confirmed Vici syndrome from whom MRI scans were available for review.

Supplemental Table 5 (ST5)

Muscle biopsy findings in *EPG5*-related Vici syndrome

Summary of findings on light microscopy, respiratory chain enzyme (RCE) studies, and electron microscopy (EM) in the 17 patients who underwent muscle biopsies. IFSV = increased fibre size variability; I/CN = increase in internalized and/or central nuclei; Vac = Vacuoles; Gly = increased glycogen; T1H/CFTD = type 1 hypotrophy and/or congenital fibre type disproportion; T1P = type 1 fibre predominance; Cor = cores; + = feature present; - = feature not present; ND = not done

Supplemental Figure 1 (SF1)

Head circumference centiles over time.

Where available measurements of birth and recent head circumference were plotted for each patient (n=13), demonstrating progressive microcephaly in all cases.

Supplemental Figure 2 (SF2)

Survival analysis

Patients with homozygous mutations died at a median age of 9 months compared to 48 months in patients with heterozygous *EPG5* mutations (p=0.046)

Supplemental Figure [Figure 4

Sural nerve biopsy abnormalities in *EPG5*-related Vici syndrome.

Semi-thin, toluidine blue stained cross section of a single peripheral sural nerve fascicle from patient 18.1, showing sub-total absence of myelinated axons (arrows) (magnification x 400). Neither actively degenerating axons nor evidence of demyelination were identified.]
