# Supplementary figures and images for: EPG5-related Vici syndrome: a paradigm of neurodevelopmental disorders with defective autophagy
Source: Brain. 2016 Feb 17;139(3):765–81. doi: 10.1093/brain/awv393 (PMC4766378; doi:10.1093/brain/awv393)

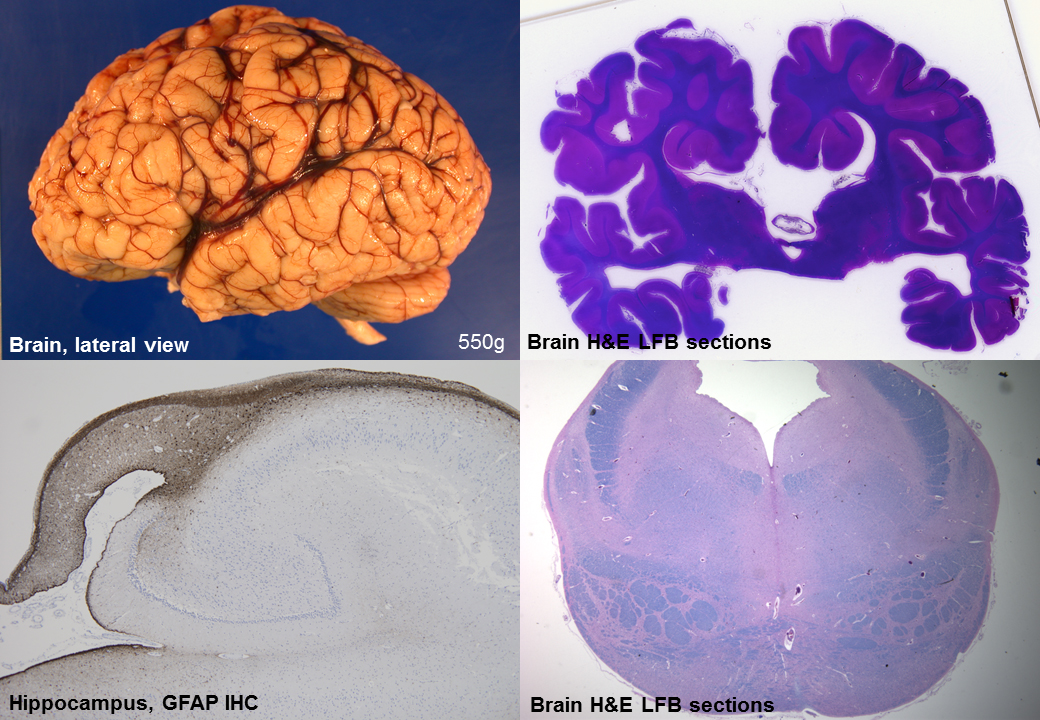

Supplement: Supplementary Data [file awv393_supplementary_data.zip › brain-2015-01466-File021.jpg]

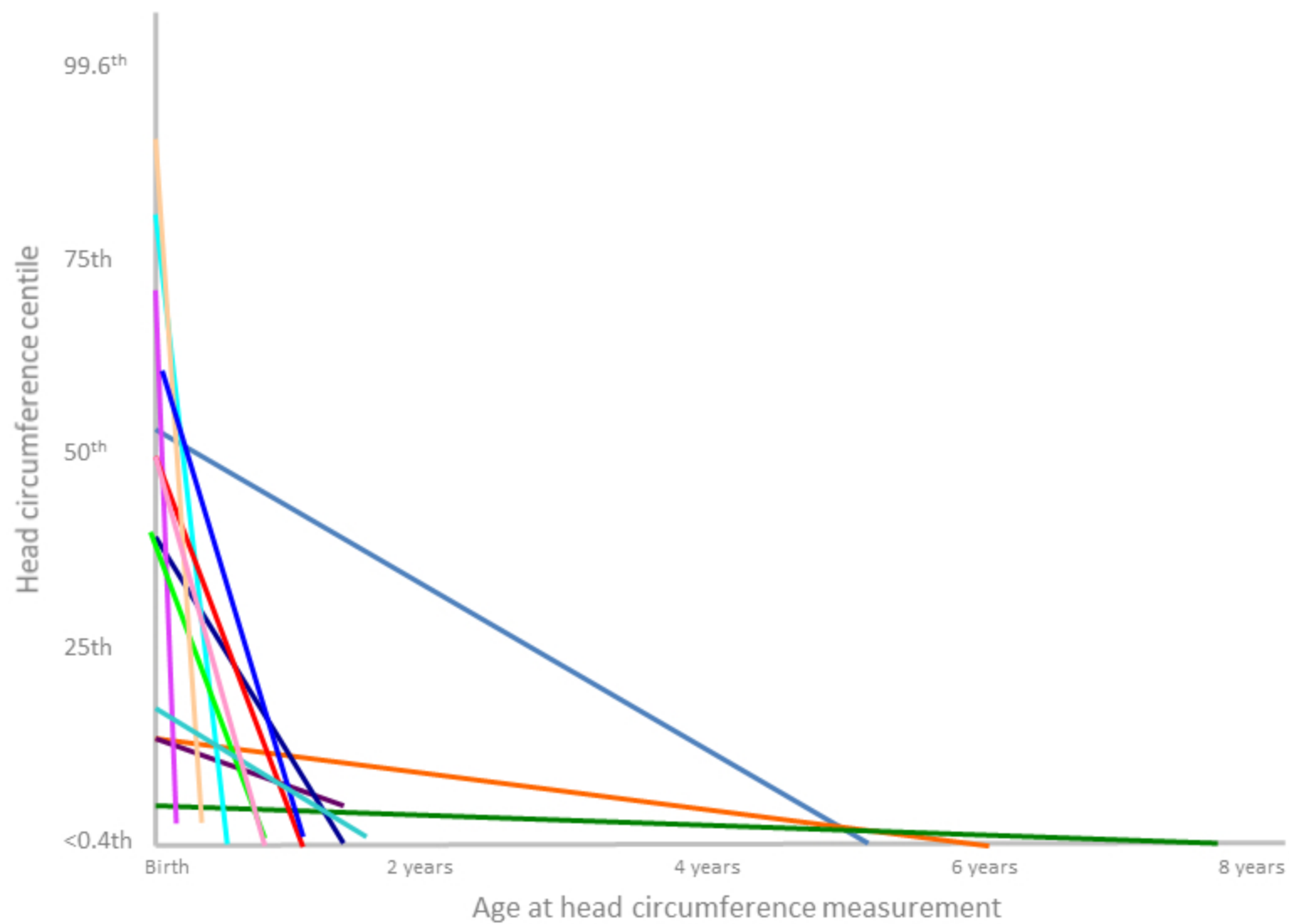

Supplement: Supplementary Data [file awv393_supplementary_data.zip › brain-2015-01466-File010.pdf]

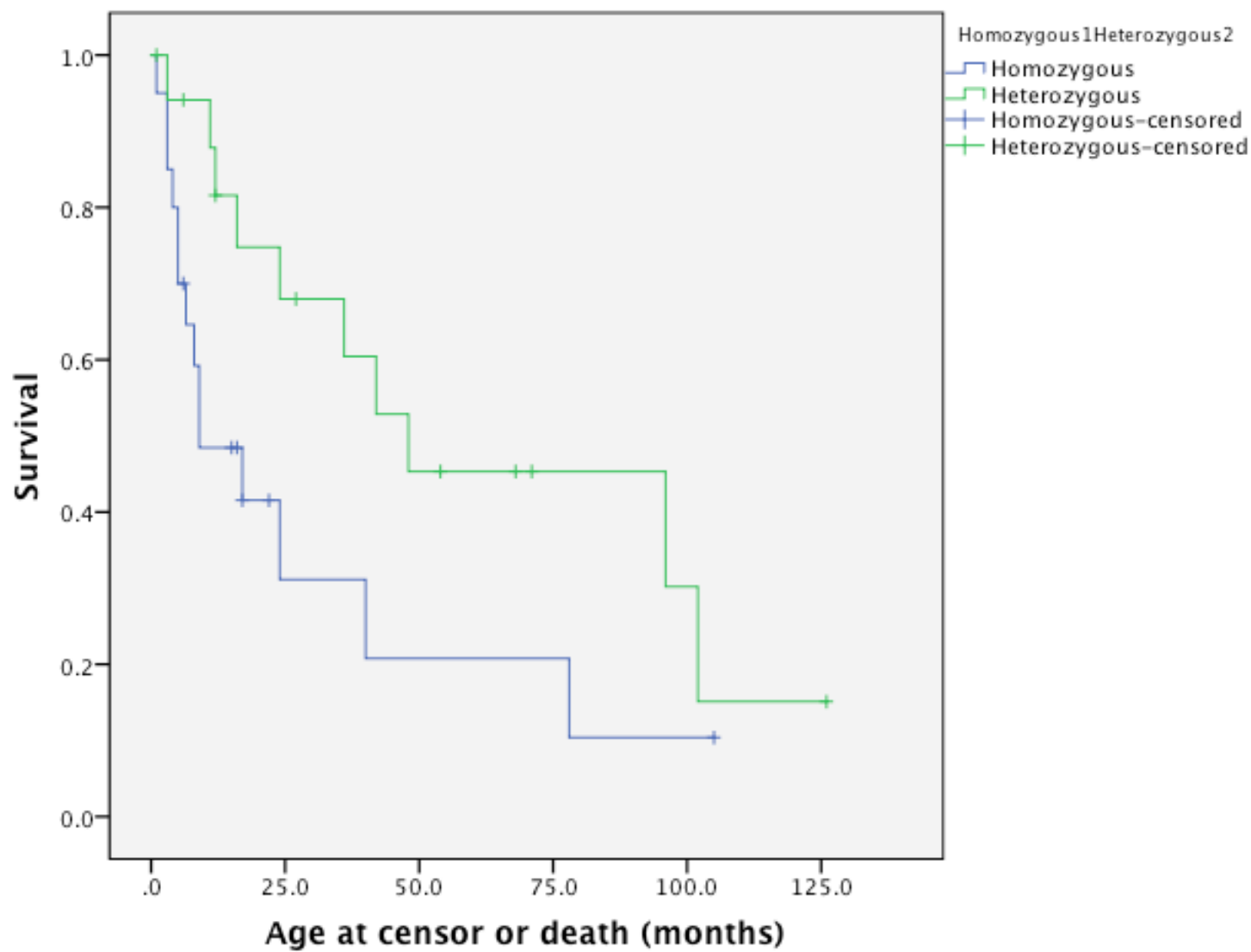

Supplement: Supplementary Data [file awv393_supplementary_data.zip › brain-2015-01466-File011.pdf]

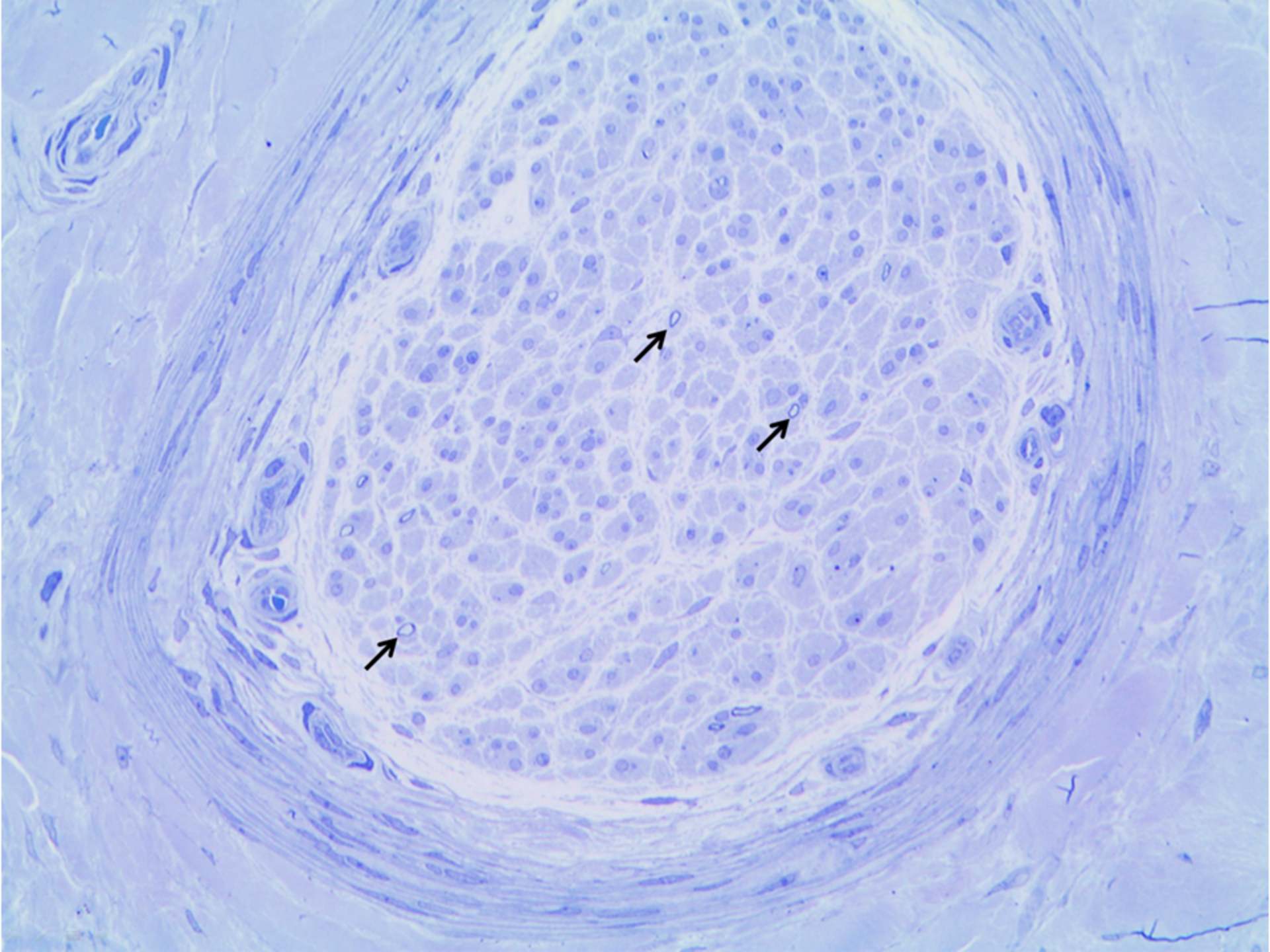

Supplement: Supplementary Data [file awv393_supplementary_data.zip › brain-2015-01466-File012.pdf]

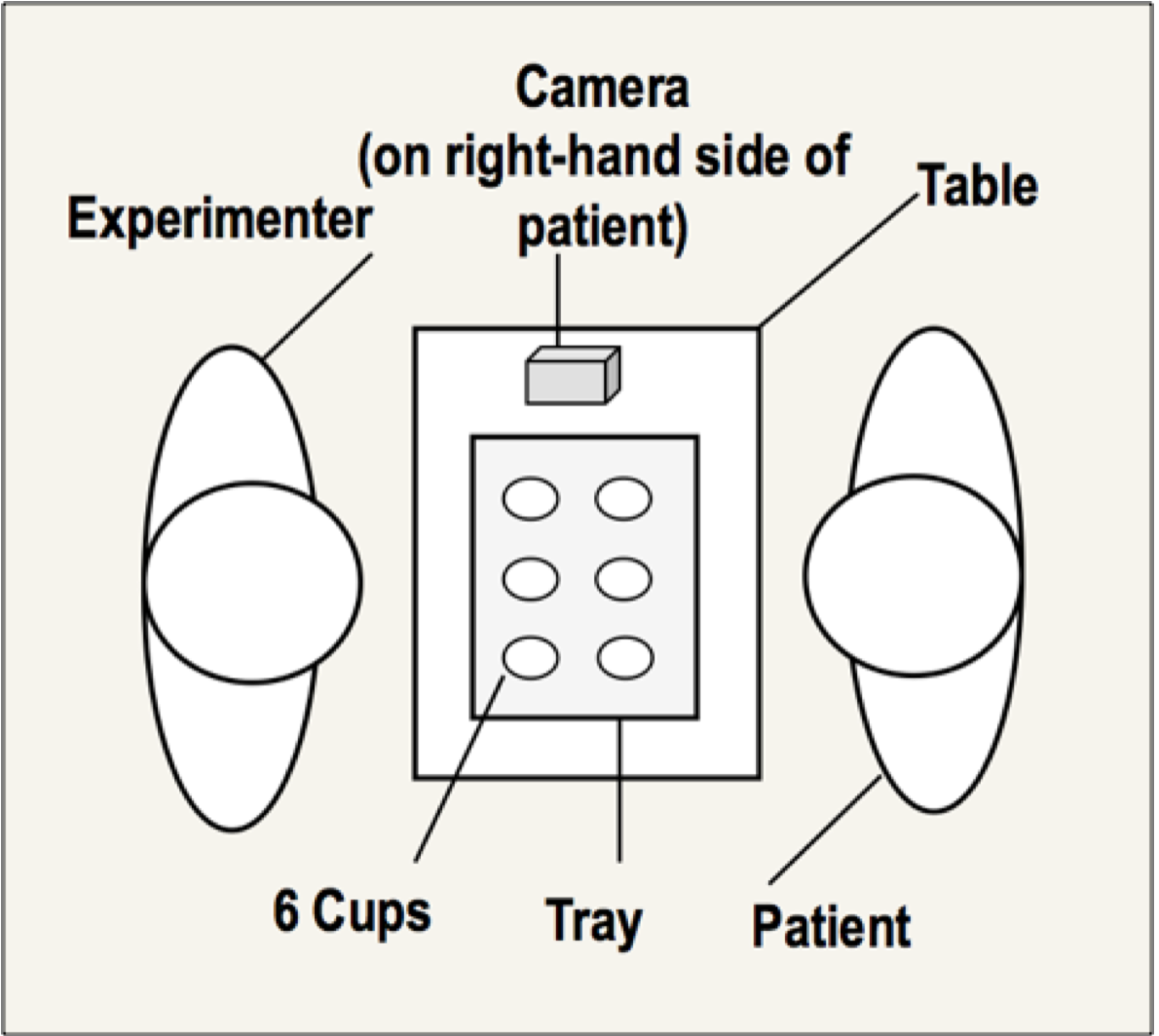

Supplement: Supplementary material [file suppl_data.zip › brain-2015-00799-File015.tiff]
